# Proteoglycan-based diversification of disease outcome in head and neck cancer patients identifies NG2/CSPG4 and syndecan-2 as unique relapse and overall survival predicting factors

**DOI:** 10.1186/s12885-015-1336-4

**Published:** 2015-05-03

**Authors:** Anna Farnedi, Silvia Rossi, Nicoletta Bertani, Mariolina Gulli, Enrico Maria Silini, Maria Teresa Mucignat, Tito Poli, Enrico Sesenna, Davide Lanfranco, Lucio Montebugnoli, Elisa Leonardi, Claudio Marchetti, Renato Cocchi, Andrea Ambrosini-Spaltro, Maria Pia Foschini, Roberto Perris

**Affiliations:** 1 Department of Biomedical and Neuromotor Sciences, Section of Anatomic Pathology, University of Bologna, Bellaria Hospital, Bologna, Italy; 2 COMT – Centre for Molecular Translational Oncology & Department of Life Sciences, University of Parma, Parma, Italy; 3 Department of Life Sciences, Division of Genetics and Environmental Biotechnology, University of Parma, Parma, Italy; 4 Department of Pathology and Laboratory Medicine, University of Parma, Parma, Italy; 5 S.O.C. of Experimental Oncology 2, The National Tumour Institute Aviano - CRO-IRCCS, Aviano, Pordenone Italy; 6 Maxillofacial Surgery Section, Head and Neck Department, University of Parma, Parma, Italy; 7 Unit of Maxillo-Facial Surgery, Department of Oral Sciences, University of Bologna, Bellaria Hospital, Bologna, Italy; 8 Department of Biomedical and Neuromotor Sciences, Unit of Maxillo-Facial Surgery, University of Bologna, S. Orsola Hospital, Bologna, Italy; 9 Unit of Maxillo-facial Surgery at Bellaria Hospital, Bologna, Italy; 10 Unit of Maxillo-facial Surgery, “Casa Sollievo della Sofferenza”, San Giovanni in Rotondo, Italy

**Keywords:** Proteoglycans, Squamous cell carcinoma, Biomarker, NG2/CSPG4, Tumour relapse

## Abstract

**Background:**

Tumour relapse is recognized to be the prime fatal burden in patients affected by head and neck squamous cell carcinoma (HNSCC), but no discrete molecular trait has yet been identified to make reliable early predictions of tumour recurrence. Expression of cell surface proteoglycans (PGs) is frequently altered in carcinomas and several of them are gradually emerging as key prognostic factors.

**Methods:**

A PG expression analysis at both mRNA and protein level, was pursued on primary lesions derived from 173 HNSCC patients from whom full clinical history and 2 years post-surgical follow-up was accessible. Gene and protein expression data were correlated with clinical traits and previously proposed tumour relapse markers to stratify high-risk patient subgroups.

**Results:**

HNSCC lesions were indeed found to exhibit a widely aberrant PG expression pattern characterized by a variable expression of all PGs and a characteristic *de novo* transcription/translation of GPC2, GPC5 and NG2/CSPG4 respectively in 36%, 72% and 71% on 119 cases. Importantly, expression of NG2/CSPG4, on neoplastic cells and in the intralesional stroma (Hazard Ratio [HR], 6.76, *p* = 0.017) was strongly associated with loco-regional relapse, whereas stromal enrichment of SDC2 (HR, 7.652, *p* = 0.007) was independently tied to lymphnodal infiltration and disease-related death. Conversely, down-regulated SDC1 transcript (HR, 0.232, *p* = 0.013) uniquely correlated with formation of distant metastases. Altered expression of PGs significantly correlated with the above disease outcomes when either considered alone or in association with well-established predictors of poor prognosis (i.e. T classification, previous occurrence of precancerous lesions and lymphnodal metastasis). Combined alteration of all three PGs was found to be a reliable predictor of shorter survival.

**Conclusions:**

An unprecedented PG-based prognostic portrait is unveiled that incisively diversifies disease course in HNSCC patients beyond the currently known clinical and molecular biomarkers.

**Electronic supplementary material:**

The online version of this article (doi:10.1186/s12885-015-1336-4) contains supplementary material, which is available to authorized users.

## Background

Head and neck squamous cell carcinomas (HNSCC) have an estimated frequency of 38,160 new cases in the US (updated to August, 2014) [1] and an estimated occurrence of more than 442,000 new cases worldwide according to GLOBOCAN 2012 [2,3], thereby representing the primary lethal cancer entity in patients with head and neck tumours. Loco-regional relapsing is the most severe clinical problem encountered in these tumours, while the pre-operative presence of lymphnodal infiltration is a recognized prognostic factor [4,5]. Especially in patients presenting smaller primary lesions, occult secondary lesions in lymphnodes significantly complicate the clinical management of these individuals [6-13]. The currently adopted methods to predict disease recurrence, such as staging and grading, are too arbitrary and do not allow for a sufficiently accurate clinical stratification of the patients [14,15]. This deficit calls upon the need to identify distinct molecular markers that more reliably would predict disease progression, recurrence and metastasis formation, and many such have been proposed over the last decade (Table 1). Thus far, however, only three such markers have been considered as meaningful, i.e. HPV infection, TP53 mutation status and overexpression of EGFR [16-20], but their full independence from clinical parameters is still dubious.

**Table 1:** **Previously proposed prognostic biomarkers in HNSCC** ^**1**^

Biomarker^2^	Clinical outcome	Method of detection	N. of cases/%/type of modulation	Annotation
**ADAM17**	Lymph nodal metastasis/Loco-regional relapse	IHC/WB	50/46/Up	None
**CD44**	OS/DFS	IHC	138/59/Down	None
***E-cadherin***	Recurrence/OS	IHC	50/20/Up 112/59/Down	None
**EGFR**	OS	IHC	109/73/Up 59/58/Up	None
**Estrogen-R2**	OS	IHC/nPCR + sequencing	67/51/Up	Laryngeal/hypopharingeal cancer
**FHIT**	OS/DFS	IHC	53/61/Down	None
**GLUT1**	OS	IHC	40/26/up	Poor radiation response
**HIF1A**	OS/DFS	IHC	85/63/Down	None
**Keratin-18**	OS	IHC	308/54/Up	None
**Keratin-8**	OS	IHC	308/54/Up	None
**Laminin** γ**2**	DSS	DNA Microarray	119/NS/Up	None
**MCM5**	OS	IHC	97/61/Up	None
**MET**	OS	IHC	69/82/Up	None
**Moesin**	OS	IHC	103/NS/Up	Cytoplasmic expression pattern
**Mucin-1**	OS/DFS/Lymphnodal metastasis	IHC	206/39/Up	Within 5-years follow-up
**Mucin-4**	OS/DFS/Lymphnodal metastasis/Loco-regional relapse	IHC	150/41/Up	Within 5-years follow-up
**p21**	OS	IHC	192/71/Down	None
**p27**	DFS	IHC	192/80/Down	Only in patients with lymphnodal infiltration
**p57**	OS	IHC	67/87/Down	None
**p63**	OS	IHC	62/NS/Up	None
**P-cadherin**	Disease recurrence/Loco-regional relapse/OS	IHC	50/20/Down 67/45/Down 108/16/Down	None
**Podoplanin**	DSS	IHC	35/56/Up	None
**Rb**	DFS	IHC	220/49/Down	Only in p53^+^/pRb^−^ patients
**RUNX3**	OS	IHC/WB	108/46/Down	None
**S100A2**	DFS/Cervical metastasis	RT-PCR + seq/IHC	135/26/Down 52/NS/Down	Nuclear expression pattern
**SPARC**	OS/DFI	DNA Microarray/IHC	62/NS/Up	None
**STAT1**	OS	IHC	89/NS/Up	None
**Survivin 3**α	OS	RT-PCR	97/NS/Up	Only in lymphnodes
**TERT**	OS	IHC	62/NS/Up	None
Ezrin	OS	IHC	47/85/Up	Cytoplasmic expression pattern

^1^Specifically referred to oral and oropharyngeal squamous cell carcinoma;

^2^Alterations of TP53, CCND1 and FGFR4 genes are not included;

*Abbreviations:***OS**, Overall Survival; **DFS**, Disease Free-Survival; **DSS**, Disease Specific-Survival; **DFI**, Disease Free-Interval; **IHC**, Immunohistochemistry; **WB**, Western Blotting; **nPCR**, nested Polymerase Chain Reaction; **NS**, Not Specified.

One class of molecules with the potential of acting as clinically relevant factors in HNSCC, especially for oral cavity and oropharynx cancer, is that comprising cell surface-associated proteoglycans (PGs). In fact, changes in their relative expression are progressively being associated with neoplastic transformation, propagation of local tumour masses, and formation of distant metastases. This not only in HNSCC, but also in numerous epithelial and non-epithelial tumour types. Both PGs produced by the HNSCC cells themselves and PGs associated with the intra-lesional tumour stroma may play critical roles in the control of HNSCC growth, dissemination and therapeutic refraction, and may therefore be contemplated as putative biomarkers as well as therapeutic targets. There are currently 15 cell surface PGs known in the human genome with the most representative ones belonging to either the transmembrane syndecan group, i.e. syndecan-1-4 (SDC1-SDC4) [21-25], or the GPI-anchored glypican group, i.e. glypican-1-6 (GPC1-GPC6) [23,26-28]. The unique structural traits of cell surface PGs enable them to modulate directly and/or indirectly several facets of the tumour cell phenotype and behavior, including growth kinetics, invasiveness and metastatic ability.

Previously documented, representative examples of the implication of diverse PGs expressions for disease outcome are afforded by the recently consolidated tumour-suppressing effect of GPC5 in lung carcinomas arising in “never smokers” [29-31], as well as by the well-established prognostic/predictive up-regulation of GPC1 in pancreatic cancer [32,33]. As a corollary, GPC3 is a recognized prognostic/predictive factor and therapeutic target in hepatocellular carcinoma [34-37]. SDC1, the only PG for which there is some documentation in oral squamous cell carcinoma, seems to be associated with the differentiation status of the tumour cells [38-40]. Clinical correlation of SDC1 expression with disease status specifically refers to its modulation in epithelial neoplastic cells [41-47] and tumour stroma [48], while the PG has been proposed to influence migration and invasion of oral squamous cell carcinoma cells *in vitro* by interacting with the β1 integrin subunit and the laminin β1 chain [48].

NG2/CSPG4 has been proposed to impact on tumourigenesis and evidence has been accrued suggesting that NG2/CSPG4 alone is able to confer metastatic potential to cancer cells by serving as a multivalent mediator of the cancer cell-host microenvironment interactions and by enhancing drug resistance and protecting cells from stress-induced programmed cell death [49,50]. In an increasing number of tumours, prognostic implications of NG2/CSPG4 are being unveiled and these discoveries accentuate the potential of the PG as a therapeutic target. Recently, a direct link between methylation and CSPG4 expression in HNSCC HPV-negative/stage IVa subgroup were proved, where high protein expression and low promoter methylation were significantly associated with an adverse progression-free and overall survival [51].

Based upon previously accrued information about the role of PGs in cancer and the currently available experimental evidences along this line, we have addressed the possibility that the pattern of expression of individual PGs, or groups of PGs, may act as either pro- or anti-tumourigenic and thereby be predictive, or indicative, of a discrete disease course in oral cavity HNSCC disease course.

## Methods

### Patients

Patients from whom surgical specimens were evaluated were treated surgically at the S. Orsola-Malpighi Hospital, at the Bellaria University Hospital in Bologna and at the Maxillo-Facial Surgery Division, Department of Head and Neck Surgery of the University of Parma. A total of 173 surgical specimens of primary oral cavity HNSCC were collected after informed consent obtained from each enrolled patients, all of them in adulthood (Additional file 1: Table S1; Additional file 2: Figure S1). Patients were referred to adjuvant radiation therapeutic treatment according to the guidelines defined by the National Comprehensive Cancer Network (NCCN) Clinical Practice (Version 2.2014; www.nccn.org). Clinical data were collected within the 2 years-post surgical follow-up every 6 months (Additional file 3; Additional file 2: Figure S1). The present study has been approved by the local ethics committees (Comitato Etico Provinciale di Parma –Parma University Hospital e Comitato Etico Provinciale di Bologna-Bologna University Hospital) and was conducted in compliance with the Helsinki Declaration’s Ethical Principles for Medical Research Involving Human Subjects.

### RNA extraction and qPCR

Total RNA from healthy specimens and 119 neoplastic specimens were extracted using Trizol® according to the manufacturer’s instructions and in combination with Qiagen RNAeasy Mini Kit (Qiagen). Total RNA (1 μg) was reverse-transcribed with the QuantiTect® Reverse Transcription Kit (Qiagen). Each TaqMan Low Density Array was designed for quantification of the human PGs. The assays were chosen among the TaqMan Gene Expression Assay library (Additional file 3) and the cards were run on ABI PRISM 7900 HT Fast Real-Time PCR System (Applied Biosystems Inc., Foster City, CA, USA). Changes in gene expression levels were calculated using the “relative quantification method”. Relative gene expression fold-change were expressed as Log_2(2^^-ΔΔCt^) and to visualize the obtained expression profiles we used heatmap graphing by EPCLUST – Expression Profile data CLUSTering and analysis software (www.bioinf.ebc.ee/EP/EP/EPCLUST/) [52]. The data presented herein have been deposited in NCBI’s Gene Expression Omnibus [53] and are accessible through GEO Series accession number GSE33788 (http://www.ncbi.nlm.nih.gov/geo/query/acc.cgi?acc=GSE33788) (Additional file 3).

### Tissue microarray (TMA) construction

Tissue specimens form a total of 163 patients, which were independently assured to contain representative areas of the neoplastic lesions, were selected for TMA construction according to a previously described procedure [54,55]. Cases were considered representative when at least 50% of the section was composed of neoplastic cells. For each case, the core portion of the section with the highest percentage of tumour cells was used for analysis (Additional file 3).

### Immunohistochemistry

Details on the antibodies used, characteristics of control tissues and experimental procedures are reported in Additional file 3. Relative antigen expression was assessed semi-quantitatively according to the arbitrary scoring: “-” = no positive cells were detected, “+” <10% of cells were positive, ≥10% “++” <50% of cells were positive, ≥50% “+++” <90% of cells were positive, and “++++” ≥90% of cells were positive.

### Statistical and bioinformatic analyses

Demographic data, presence of recognized risk factors for development of HNSCC, clinical diagnostic parameters, gene expression and protein distribution patterns for the PGs GPC1-6, SDC1-4 and NG2/CSPG4 were comparatively evaluated for their potential correlation with the following disease outcomes: loco-regional recurrence, lymphnodal metastasis, distant metastasis, disease-related deaths and probability of incurring into one or more of these clinical outcomes. Estimation of influence of each variable considered for the above disease outcomes was analyzed independently with both the Log-rank and Wilcoxon’s rank test. Survival rate was estimated using the Kaplan-Meier method from the time of surgery to the end of the follow-up. Cox’s multivariate proportional hazards regression method was used to extract a parsimonious set of independent variables. All analyses were performed using the Statgraphics Centurion XVI software (StatPoint Technologies, Inc, Virginia, USA). *P* values <0.05 were considered to be significant (Additional file 3).

## Results

### Transcriptional profiles of PGs in primary oral cavity HNSCC lesions

**Figure 1:**
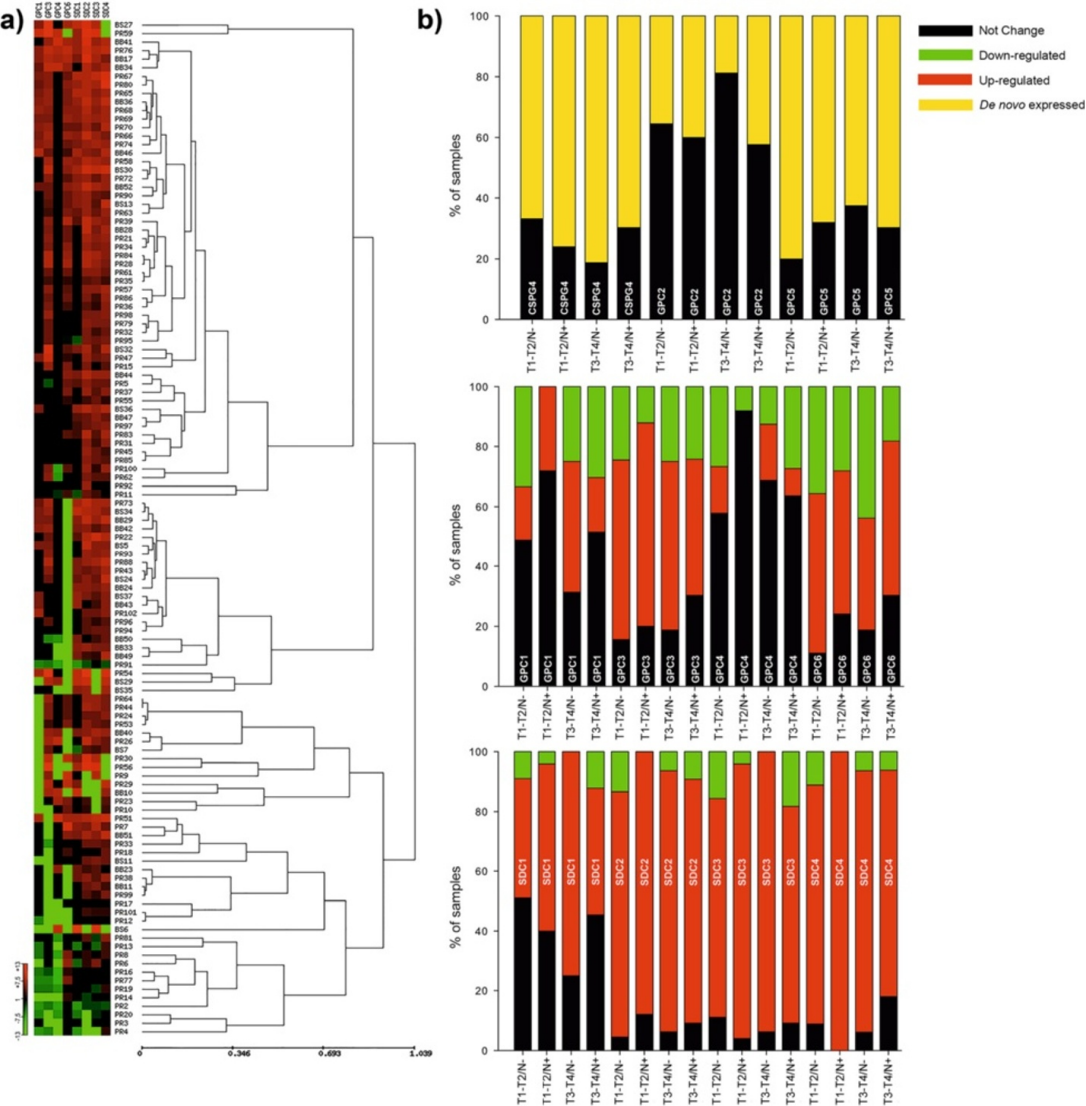
Cell surface-associated PGs are differentially expressed in primary lesions of oral cavity HNSCC patients. **(a)** Heat map and hierarchical clustering of the relative expression levels (columns) of the 8 most modulated cell surface-associated PGs in primary lesions of 119 oral cavity HNSCC patients (*rows*): *red*, up-regulation; *green*, down-regulation, *black*: no change in comparison to the healthy epithelial tissue. Distance between clusters was calculated as reported in Additional file 3. **(b)** PG expression profiles in oral cavity HNSCC specimens derived from T1-T2-N-, T1-T2-N+, T3-T4-N- and T3-T4-N+ graded tumours. SDC1-4, syndecans 1–4; GPC1-6, glypicans 1–6.

Analyses of the relative mRNA expression levels of the eleven prevalent cell surface-associated PGs conducted on a total of 119 primary oral cavity HNSCC lesions revealed that 3 of the PGs, including NG2/CSPG4, GPC2 and GPC5, were *de novo* expressed in neoplastic cells, i.e. were not detectable in the healthy control tissues, but were detectable in cancer cells. These were transcribed in 71% (NG2/CSPG4), 36% (GPC2) and 72% (GPC5) of the lesions, respectively. The remaining 8 PGs, for which transcripts were expressed at a frequency of 84% (GPC1), 86% (GPC3), 88% (GPC4), 70% (GPC6), 100% (SDC1), 94% (SDC2), 93% (SDC3) and 95% (SDC4) of the tumour cases, respectively, were found to be differently modulated. Thus, SDC2, SDC3 and SDC4 were up-regulated in 79-84% of the patients, whereas GPC4 was enhanced in 11% and GPC3 in 57% of the specimens. However, GPCs were more frequently down-regulated (GPC3, 22%; GPC4, 21%; GPC1, 24%; and GPC6 30%) than SDCs (SDC1, 8%; SDC2, 8%; SDC3, 11%; and SDC4, 7%; Figure 1a; Additional file 4: Table S2).

We next compared the PG expression patterns exhibited by discrete groups of patients differentiated by tumour staging, i.e. T1-T2/N- versus T1-T2/N+ and T3-T4/N- versus T3-T4/N+. GPC2 was expressed in a mere 19% of the T3-T4/N- classified lesions, whereas it was a two-fold more frequently transcribed in patients belonging to the other three classes (T1-T2/N-, 36%, T1-T2/N+, 40% and T3-T4/N+, 42%). GPC5 and NG2/CSPG4 were detectable in samples of a large proportion of patients, ranging from 62% in T3-T4/N- to 80% in T1-T2/N-, but their relative expression levels did not discriminate between the above patient subsets (Figure 1b). GPC1 was similarly differently expressed in the distinct groups of patients with a 1.5-fold higher frequency in the T3-T4/N- patients compared to the other patient subsets. GPC4 was down-regulated in about 27% of the T1-T2/N- and T3-T4/N+ subgroups, and <13% in the T1-T2/N+ and T3-T4/N- patient subgroups (Figure 1b). Syndecans were generally up-regulated in most of the lesions (Figure 1b), with SDC4 showing enhanced expression in 100% of T1-T2/N+ patients.

### Immunolocalization of PGs in oral cavity HNSCC lesions

**Figure 2:**
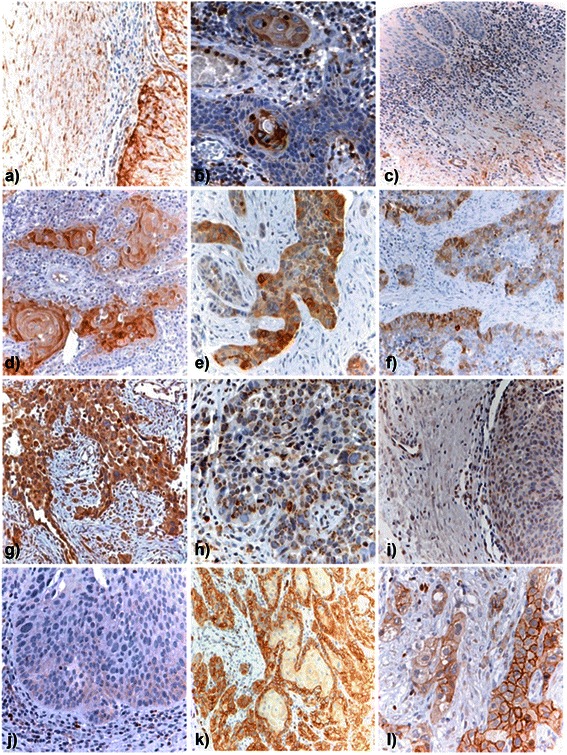
In situ immunolocalization of GPCs and NG2/CSPG4 in oral cavity HNSCC primary lesions. Representative patterns of GPC and NG2/CSPG4 distribution in oral cavity HNSCC lesions. **(a**, **b)** representative views of GPC1 expression in lesions with different degrees of keratinizing neoplastic cells. **(c**, **d)** representative images of GPC1 expression in stromal cells of pre-malignant lesions **(c)** and lack of expression in the stromal cells of HNSCC tissue **(d)**. GPC3 was detected in neoplastic cells **(e)**, but not stromal fibroblasts **(f)**, whereas GPC4 **(g)** and GPC6 **(h)** were primarily found to be associated with the neoplastic cells. **(i)** Shows the lack of expression of GPC6 in the intralesional stroma. NG2/CSPG4 was found to be abundantly expressed in both well- **(k)** and moderately-differentiated **(l)** oral cavity HNSCC lesions, whereas it was similarly absent from potentially pre-malignant lesions **(j)**.

**Figure 3:**
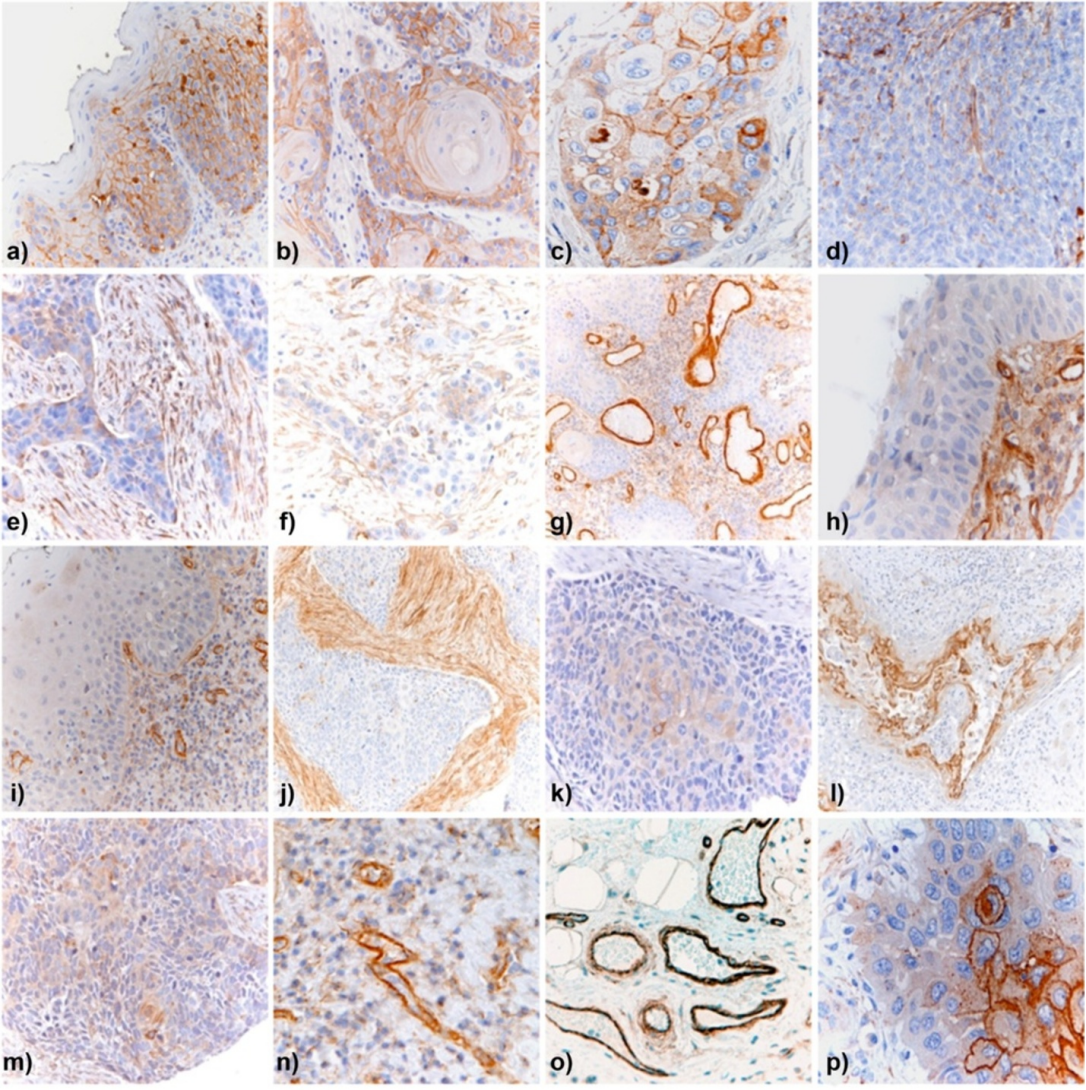
Immunodetection of SDCs in oral cavity HNSCC primary lesions. Representative view of the SDC1 expression pattern, inversely correlating with the overall differentiation status of the tumour (**a**, displatyc tissue; **b**, **c**, well-differentiated; **d**, poorly differentiated), while being particularly abundant in the center of neoplastic nests **(p)** and in the stromal compartment **(e-f)**. SDC2 was seen strongly associated with tumour vessels **(g, n, o)** and was the only PG to be widely expressed in the different degree of dysplastic tissue **(h-j)**. SDC3 (**k**) and SDC4 **(m)** immunolocalized in the epithelial tumour cells, but not in the stromal compartment (**l**, SDC3; **m**, SDC4).

Intralesional distribution of PGs was further examined in oral cavity HNSCC lesions and control healthy tissue using empirically validated, pre-selected antibodies against each of the PGs (Figure 2; Figure 3; Additional file 5: Figure S2). The percentage of cases in which GPCs could be disclosed on the epithelial neoplastic cells varied from 18% (30 out of 163 cases) for GPC3 to 72% for GPC1 (118 out of 163 cases; Table 2). Relative frequency of expression was in the order: GPC1 > GPC4 (41%; 67 out of 163 cases) > GPC6 (37%; 61 out of 163 cases) > GPC3. GPCs were often detected within the cytoplasm as well as on the cell membrane, consistent with their thoroughly described internalization and recycling patterns. The hybrid cell membrane/cytoplasmic distribution of these PGs was characteristically observed for GPC1, GPC3 and GPC4, with GPC1 being most strongly associated with these two cellular compartments in keratinizing neoplastic cells (Additional file 5: Figure S2). GPC3 was entirely absent in healthy tissue, while GPC4 showed a widespread distribution both on normal epithelial cells and in the intralesional stromal compartment (Additional file 5: Figure S2). GPC6 appeared to be preferentially retained within intracellular vesicles (Figure 2), as deduced by the appearance of GPC6-positive granules throughout the cytoplasm of neoplastic cells. This seemed rather specific for tumour cells since it was not observed in healthy epithelial cells (Additional file 5: Figure S2). GPC1 and GPC6 were rarely seen in the intralesional stroma of oral cavity HNSCC lesions (13 out of 163 and 16 out of 163 of the cases, respectively), whereas GPC3 was consistently absent from this compartment and GPC4 showed a somewhat more frequent expression in stromal cells (19%; 31 out of 163 of the cases; Table 2; Figure 2).

**Table 2:** **Patterns of the in situ distribution of PGs in HNSCC lesions (% of cases)**

PG	Tumor cell positivity^1^		Overall staining intensity^2^		Subcellular localization^3^		Stromal expression	
GPC1	-	(27.5)	-	(27.6)	Membrane	-	-	(92.0)
	+	(29.2)	+	(33.1)	Cytoplasmic	-	+	(6.7)
	++	(33.1)	++	(30.1)	Both	(72.4)	++	(0.6)
	+++	(7.4)	+++	(6.7)			+++	(0.6)
	++++	(2.5)	++++	(2.5)			++++	-
GPC3	-	(81.6)	-	(81.6)	Membrane	-	-	(100.0)
	+	(14.1)	+	(14.1)	Cytoplasmic	-	+	-
	++	(3.1)	++	(4.3)	Both	(18.4)	++	-
	+++	(1.2)	+++	-			+++	-
	++++	-	++++	-			++++	-
GPC4	-	(58.9)	-	(59.5)	Membrane	-	-	(81.0)
	+	(20.9)	+	(14.1)	Cytoplasmic	(40.5)	+	(5.5)
	++	(19.0)	++	(25.8)	Both	-	++	(12.9)
	+++	(1.2)	+++	(0.6)			+++	(0.6)
	++++	-	++++	-			++++	-
GPC6	-	(62.2)	-	(62.6)	Membrane	-	-	(90.2)
	+	(30.1)	+	(31.9)	Cytoplasmic	(37.4)	+	(9.8)
	++	(7.4)	++	(5.5)	Both	-	++	-
	+++	-	+++	-			+++	-
	++++	-	++++	-			++++	-
SDC1	-	(8.6)	-	(8.6)	Membrane	(42.9)	-	(79.1)
	+	(31.3)	+	(25.8)	Cytoplasmic	(37.4)	+	(9.2)
	++	(33.7)	++	(50.3)	Both	(10.4)	++	(10.4)
	+++	(22.1)	+++	(10.4)			+++	(1.2)
	++++	(4.3)	++++	(4.9)			++++	-
SDC2	-	(88.3)	-	(88.3)	Membrane	(1.8)	-	(26.4)
	+	(6.7)	+	(8.0)	Cytoplasmic	(8.6)	+	(20.2)
	++	(4.3)	++	(3.1)	Both	-	++	(25.8)
	+++	(0.6)	+++	(0.6)			+++	(21.5)
	++++	-	++++	-			++++	(6.1)
SDC3	-	(65.0)	-	(65.0)	Membrane	-	-	(100.0)
	+	(27.0)	+	(28.2)	Cytoplasmic	-	+	-
	++	(6.1)	++	(6.7)	Both	(34.4)	++	-
	+++	(1.8)	+++	-			+++	-
	++++	-	++++	-			++++	-
SDC4	-	(80.4)	-	(80.4)	Membrane	-	-	(100.0)
	+	(16.0)	+	(19.6)	Cytoplasmic	-	+	-
	++	(3.7)	++	-	Both	(17.8)	++	-
	+++	-	+++	-			+++	-
	++++	-	++++	-			++++	-
NG2/CSPG4	-	(34.2)	-	(34.2)	Membrane	(62.6)	-	(96.7)
	+	(38.8)	+	(36.8)	Cytoplasmic	-	+	(3.3)
	++	(21.7)	++	(19.7)	Both	-	++	-
	+++	(2.6)	+++	(7.2)			+++	-
	++++	(2.6)	++++	(2.0)			++++	-

^1^PG expression was assessed semi-quantitatively according to the arbitrary scoring: “-”, no positively staining cells were detected; “+”, <10% of cells were positive; “++”, ≥10% and <50% of positive cells; “+++”, ≥50% and <90% of positive cells; “++++”, ≥90% of positive cells;

^2^Refers to the average staining intensity within the examined lesion, according to the arbitrary scoring: “-” = absent; “+”, faint; “++”, weak; “+++”, moderate; “++++”, strong;

^3^Immunostaining was prevalently cell membrane-associated (“Membrane”) or diffuse cytoplasmatic (“Cytoplasmic”).

HNSCC lesions showed variable expression of SDCs with a relative frequency of positive cases decreasing in the order: SDC1 > SDC3 > SDC4 > SDC2 (Table 2). In fact, a total of 149 cases out of 163 lesions that were evaluated for the *in situ* expression of the SDC1/CD138 protein had epithelial neoplastic cells presenting the PG on the cell surface, or in intracellular locations (Figure 3). In contrast, a mere 57 (35%), 32 (19.6%), and 19 (12%) out of 163 examined lesions had epithelial neoplastic cells staining positively for respectively SCD2, SDC3 and SDC4 (Table 2). The relative number of cancer cells that expressed these PGs in each lesion markedly differed and a similar divergence was seen in terms of subcellular localization of the molecules. Thus, neoplastic cells with plasma membrane-associated SDC1 were mainly keratinizing cells located at the center of the neoplastic nests (Figure 3). Fibroblasts of the tumour stroma that surrounded the neoplastic nests were positive for SDC1 in 34 out of 163 (21%) of the tumours. In 14 out of 163 of the lesions (8.6%), SDC2 was immunolocalized within the cytoplasm of neoplastic cells, while it appeared widespread in the stromal cells of the majority of the lesions (73.6%, 120 out of 163 of the cases; Figure 3) and was particularly enriched in lesions containing desmoplastic stroma. Intriguing was the fact that in 100% of the lesions, SDC2 could be observed in the wall of both normal and intra-lesional blood vessels, suggesting that it was associated with both endothelial and neovascular pericytes (Figure 3). In contrast to SDC1 and SDC2, both SDC3 and SDC4 were undetectable in the healthy epithelium, or the tumour stroma, but could be immunolocalized both subcellularly and on defined portions of the cell surface of neoplastic cells, with a particular concentration in focal plaque-like structures (Figure 3; Additional file 5: Figure S2). Finally, the diversity of SDCs expressions in oral cavity HNSCC lesions was even more remarkable when considering the relative distribution of these PGs in the stromal compartment. In this case, the frequency of occurrence of the PGs was largely reversed with respect to that seen in the cancer cells and decreased in the order: SDC2 > SDC1 > SDC3 = SDC4 (Table 2). Deviating from the pronounced intracellular distribution of SDC3 and SDC4 was that of NG2/CSPG4 which showed an exclusive cell membrane localization in all samples in which the PG could be disclosed (63%). NG2/CSPG4 was rarely detected in the stromal compartment (5 out of 163; Table 2; Figure 2), where, if occurring, was concentrated on the membrane of basal cells (Additional file 5: Figure S2).

### Altered expression of discrete PGs correlates with disease outcome

All demographic and clinical-pathological traits of the patients were initially compared by univariate analysis of the cumulative PG expression data, except for patient categories comprised of less than 13 patients (independently defined as a cut-off level of “statistical” exclusion). In these correlation analyses we considered five primary disease outcomes, including loco-regional tumour recurrence, lymphnodal metastasis, distant metastases, disease-related death and a situation in which at least one of the former disease outcomes was manifested (Table 3).

**Table 3:** **Univariate analysis of PG expression in relation to known prognostic indicators**

Prognostic indicator/PG			N_._of cases	Clinical outcomes									
				Loco-regional recurrence		Lymphnodal metastasis		Distant metastasis		Disease-related death		Any of the clinical outcomes^1^	
				% cases	*p value*	% cases	*p value*	% cases	*p value*	% cases	*p value*	% cases	*p value*
*Prognostic indicator*													
Sex		Male	99	14.1	0.342	15.2	**0.043**	10.1	0.847	22.2	0.155	36.4	0.094
		Female	74	7.0		5.4		9.5		13.5		24.3	
Age		≤45 yrs	17	11.8	0.689	11.8	0.964	0.0	-	5.9	0.146	17.6	0.184
		>45 yrs	156	12.2		10.9		10.9		19.9		32.7	
Smoking		No	66	13.6	0.689	9.1	0.466	9.1	0.718	12.1	0.109	25.8	0.253
		Yes	105	11.4		12.4		10.5		21.9		34.3	
Alcohol^2^		No	83	10.8	0.521	8.4	0.231	4.8	**0.026**	6.0	**<0.001**	21.7	**0.012**
		Yes	88	13.6		13.6		14.8		29.5		39.8	
Familial cancer history		No	144	12.5	0.732	10.4	0.615	9.7	0.880	18.8	0.876	31.3	0.994
		Yes	27	11.1		14.8		11.1		18.5		33.3	
Precancerous lesions		No	121	12.4	0.869	5.8	**0.003**	9.9	0.970	18.2	0.869	28.1	0.305
		Yes	51	11.8		21.6		9.8		19.6		37.3	
Tumor site		OC	156	12.2	0.530	11.5	0.204	10.3	0.776	17.9	0.858	31.4	0.399
		OP	14	7.1		0.0		7.1		21.4		21.4	
		OC+OP	3	33.3		33.3		0.0		33.3		66.7	
T classification		T1	51	3.9	**0.007**	7.8	0.326	2.0	0.073	5.9	**0.038**	17.6	**0.018**
		T2	60	8.3		8.3		16.7		21.7		30.0	
		T3	17	23.5		17.6		5.9		23.5		47.1	
		T4	45	22.2		15.6		11.1		26.7		42.2	
		T1-T2	111	6.3	**0.001**	8.1	0.065	9.9	0.957	14.4	**0.043**	24.3	**0.005**
		T3-T4	62	22.6		16.1		9.7		25.8		43.5	
N classification		Negative	97	9.3	0.079	5.2	**0.001**	5.2	**0.016**	9.3	**<0.001**	19.6	**<0.001**
		Positive	76	15.8		18.4		15.8		30.3		46.1	
Differentiation Degree		Well	20	15.0	0.441	0.0	0.223	5.0	0.392	15.0	0.952	20.0	0.44
		Moderate	50	16.0		12.0		14.0		18.0		36.0	
		Poor	78	7.7		12.8		7.7		16.7		30.8	
Radiotherapy		No	83	8.4	0.082	4.8	**0.005**	3.6	**0.007**	8.4	**<0.001**	20.5	**0.002**
		Yes	90	15.6		16.7		15.6		27.8		41.1	
*PG (mRNA)* ^3^													
SDC1		↓ / =	61	11.5	0.553	6.6	0.396	4.9	**0.016**	11.5	**0.036**	21.3	**0.013**
		↑	58	13.8		10.3		19.0		25.9		41.4	
SDC2		↓ / =	19	5.3	0.253	5.3	0.519	5.3	0.329	10.5	0.314	15.8	0.104
		↑	100	14.0		9.0		13.0		20.0		34.0	
SDC3		↓ / =	24	8.3	0.405	8.3	0.896	4.2	0.193	20.8	0.855	25.0	0.352
		↑	95	13.7		8.4		13.7		17.9		32.6	
SDC4		↓ / =	19	5.3	0.253	5.3	0.519	5.3	0.329	15.8	0.780	26.3	0.601
		↑	100	14.0		9.0		13.0		19.0		32.0	
GPC1		=	62	12.9	0.803	6.5	0.502	12.9	0.668	17.7	0.940	32.3	0.625
		↓ / ↑	57	12.3		10.5		10.5		19.3		29.8	
GPC2		=	76	10.5	0.393	6.6	0.363	10.5	0.596	21.1	0.335	27.6	0.307
		*De novo*	43	16.3		11.6		14.0		14.0		37.2	
GPC3		↓	26	7.7	0.437	0.0	0.219	11.5	0.378	11.5	0.303	23.1	0.624
		=	25	20.0		12.0		4.0		12.0		32.0	
		↑	68	11.8		10.3		14.7		23.5		33.8	
GPC4		=	81	11.1	0.598	7.4	0.647	13.6	0.365	21.0	0.308	33.3	0.348
		↓ / ↑	38	15.8		10.5		7.9		13.2		26.3	
GPC5		=	33	12.1	0.995	9.1	0.851	6.1	0.241	15.2	0.609	30.3	0.986
		*De novo*	86	12.8		8.1		14.0		19.8		31.4	
GPC6		↓	36	19.4	0.351	8.3	0.994	8.3	0.495	16.7	0.838	36.1	0.824
		=	24	8.3		8.3		8.3		16.7		29.2	
		↑	59	10.2		8.5		15.3		20.3		28.8	
NG2/CSPG4		=	34	2.9	**0.029**	8.8	0.914	8.8	0.487	14.7	0.401	20.6	0.077
		*De novo*	85	16.5		8.2		12.9		20.0		35.3	
*PG (Protein)* ^4^													
SDC1 Tumor cells		Negative	14	7.1	0.447	21.4	0.312	7.1	0.769	7.1	0.269	35.7	0.947
		Positive	149	13.4		10.7		9.4		19.5		31.5	
Stroma		Negative	129	13.2	0.962	11.6	0.812	9.3	0.998	17.1	0.294	31.0	0.429
		Positive	34	11.8		11.8		8.8		23.5		35.3	
SDC2 Tumor cells		Negative	143	13.3	0.783	11.9	0.875	8.4	0.303	18.9	0.792	30.8	0.385
		Positive	19	10.5		10.5		15.8		15.8		42.1	
Stroma		Negative	43	9.3	0.277	2.3	**0.015**	2.3	0.062	2.3	**0.001**	14.0	**0.002**
		Positive	120	14.2		15.0		11.7		24.2		38.3	
SDC3 Tumor cells		Negative	106	13.2	0.923	12.3	0.793	8.5	0.655	17.9	0.758	30.2	0.575
		Positive	56	12.5		10.7		10.7		19.6		35.7	
SDC4 Tumor cells		Negative	131	15.3	0.071	13.0	0.329	9.2	0.957	17.6	0.588	32.8	0.649
		Positive	32	3.1		6.3		9.4		21.9		28.1	
GPC1 Tumor cells		Negative	44	11.4	0.653	15.9	0.340	4.4	0.214	15.6	0.602	31.8	0.825
		Positive	108	13.6		10.2		10.7		18.9		32.2	
Stroma		Negative	149	11.4	**0.007**	12.1	0.884	8.4	0.319	16.8	**0.012**	30.9	0.104
		Positive	13	30.8		7.7		15.4		38.5		46.2	
GPC3 Tumor cells		Negative	133	12.8	0.805	12.0	0.841	9.6	0.584	18.4	0.834	33.1	0.605
		Positive	30	13.3		10.0		6.3		15.6		26.7	
GPC4 Tumor cells		Negative	96	13.5	0.969	10.2	0.493	9.2	0.928	17.3	0.716	33.3	0.794
		Positive	66	12.1		13.0		8.7		18.8		30.3	
Stroma		Negative	131	13.0	0.860	11.5	0.692	9.2	0.900	18.3	0.771	32.8	0.810
		Positive	31	12.9		12.9		9.7		19.4		29.0	
GPC6 Tumor cells		Negative	101	12.9	0.860	6.9	**0.010**	5.9	0.052	15.8	0.222	27.7	0.102
		Positive	61	13.1		19.7		14.8		23.0		39.3	
Stroma		Negative	146	11.6	0.058	12.3	0.599	10.3	-	18.5	0.844	32.2	0.828
		Positive	16	25.0		6.3		0.0		18.8		31.3	
NG2/CSPG4 Tumor cells		Negative	52	15.4	0.665	9.6	0.406	9.6	0.923	17.3	0.563	34.6	0.967
		Positive	100	12.0		14.0		10.0		21.0		33.0	
T group/NG2/CSPG4 mRNA^5^	T1-T2/=		21	0	**<0.001**								
	T1-T2 / D*e novo*		49	6.1									
	T3-T4/=		13	7.7									
	T3-T4/*De novo*		36	30.6									
Precancerous lesions/SDC2 stroma	-/-		29			0	**0.001**						
	-/+		84			8.3							
	+/-		14			7.1							
	+/+		35			28.6							
N status^6^/SDC1 mRNA	Negative/↓/=		31					0	**0.004**				
	Negative/↑		30					10.0					
	Positive/↓/=		30					10.0					
	Positive/↑		28					28.6					
N status/SDC2 stroma	-/-		33							0	**<0.001**		
	-/+		57							14.0			
	+/-		10							10.0			
	+/+		63							33.3			
N status/SDC1 mRNA	Negative/↓/=		31									3.2	**<0.001**
	Negative/↑		57									33.3	
	Positive/↓/=		10									40.0	
	Positive/↑		63									50.0	
PGs pattern													
SDC1 mRNA ↑ NG2/CSPG4 mRNA d*e novo +* SDC2 stroma	Yes		36	16.7	0.271	11.1	0.405	25	**<0.001**	33.3	**0.002**	50	**<0.001**
	No		73	12.3		8.2		4.1		11		23.3	

^1^This refers to the situation in which patients manifested at least one of the four adopted clinical outcomes within the follow-up period;

^2^Excessive alcohol consumption was based upon self-provided information;

^3^PG transcript expression was defined as “↓”, down-regulated; “↑”, up-regulated; “=”, not changed; and “*De novo”*, *de novo* expressed, when compared to a healthy mucosal tissues pool that was used as sample calibrator;

^4^Protein expression data are reported as detectable or non-detectable by indirect immunohistochemistry;

^5^Univariate analyses combining the prognostic indicators that were deemed to be independent poor predictors of each of the five clinical outcomes as accomplished through the Cox proportional hazard model;

^6^ N status positive or negative is according to N classification AJCC staging system;

*p* values <0,05 were considered to be significant (in bold); *p* values within ≥ 0,05 and <0,06 were considered borderline and were included in the following multivariate regression model; *p* value was not calculated where a monotone likelihood was established.

*Abbreviations*: OC, oral cavity; OP, oropharynx.

In order to test whether there is a relationship between PGs transcript and protein expressions and clinicopathological parameters, a Chi-Square test was applied and just three such correlation resulted statistically significant: N classification and SDC2 stromal positivity, *p* = 0.002; alcohol consumption and SDC1 mRNA up-regulation, *p* = 0.021, and presence of precancerous lesion and SDC1 mRNA up-regulation, *p* = 0.016.

Although radiation therapy and excessive alcohol consumption independently correlated with one or more of the above clinical outcomes (Table 3), these parameters were not considered in the multivariate logistic regression analyses because of being potentially confounding indicators. The first because almost all patients presenting lymphnodal infiltrations had been routinely subjected to radiation therapy, the second because, despite of its well-recognized importance as a risk factor in HNSCC, the admission of this habit was measured by a self-provided questionnaire and no details were available on the accuracy of the information provided by the patients. A further consideration is that self-reported excessive alcohol intake is often denied, causing underestimation of the cohort of patient that may fall under this “risk category”.

**Figure 4:**
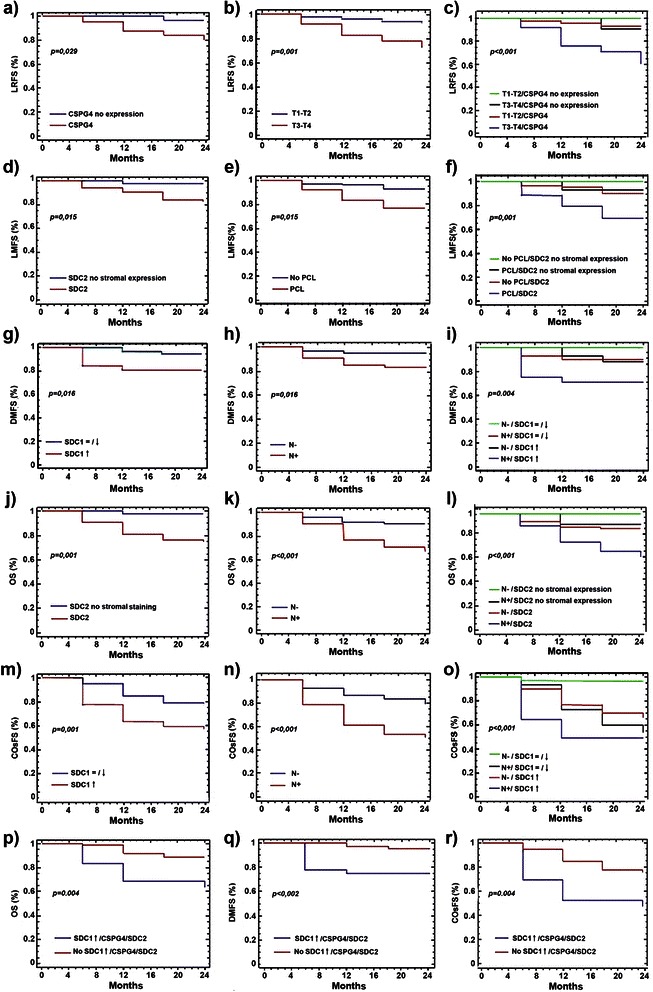
Differential PG expression correlates with clinical outcome. Survival and probability curves for the following correlations: **(a)** loco-regional relapse *vs de novo* expression of NG2/CSPG4, **(b)** loco-regional relapse *vs* T group classification; **(c)** loco-regional relapse *vs* coincident NG2/CSPG4 expression and advanced T classification; **(d)** lymphnodal metastases *vs* enhanced SDC2 expression in stromal cells; **(e)** lymphnodal metastases *vs* manifestation of precancerous lesions; **(f)** lymphnodal metastases *vs* the combination of both previous prognostic indicators; **(g)** distant metastases *vs* up-regulated SDC1 expression; **(h)** distant metastases *vs* infiltration of cervical lymph nodes; **(i)** distant metastases *vs* the combination of both previous prognostic indicators; **(j)** overall survival *vs* enhanced SDC2 expression in the stromal compartment; **(k)** overall survival *vs* N classification; **(l)** overall survival *vs* the coincidence of both previously indicated events; **(m)** the occurrence of any of the clinical outcomes *vs* up-regulated SDC1 expression; **(n)** the occurrence of any of the clinical outcomes *vs* N classification; **(o)** the occurrence of any of the clinical outcomes *vs* the combination of both above listed events; **(p)** overall survival, **(q)** distant metastases and **(r)** the occurrence of any of the clinical outcomes *vs* the combination of SDC1 up-regulation, *de novo* expression of NG2/CSPG4 and stromal enhancement of SDC2 positivity. *Abbreviations*: LRFS, loco-regional relapse-free survival; LMFS, lymphnodal metastasis-free survival; DMFS, distant metastasis-free survival; OS, overall survival, COsFS, clinical outcomes-free survival.

**Figure 5:**
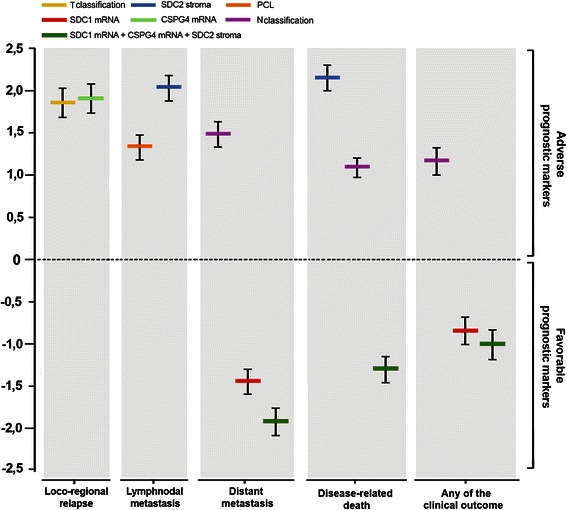
Cox proportional hazard analysis. Plot overview of Cox proportional hazard estimated regression coefficients of the resulting independent prognostic factors for loco-regional recurrence, lymphnodal metastases, distant metastases, disease-related death and the occurrence of any of the clinical outcomes.

Advanced T classification (*p* = 0.007), T3-T4 grouping (*p* = 0.001; Figure 4), positive NG2/CSPG4 transcript expression (*p* = 0.029; Figure 4), or GPC1 positivity in stromal cells (*p* = 0.007) were all conditions strongly associated with a high loco-regional tumour relapse rate (Table 3). Stromal GPC6 expression could, however, not be included as a parameter in the multivariate logistic regression model due to the low number of cases contained within this category and the borderline statistical significance in univariate analyses (*p* = 0.058). Application of the Cox proportional hazard model revealed that T3-T4 classification of the tumour (HR, 6.36, *p* = 0.001) and *de novo* expression of NG2/CSPG4 mRNA (HR, 6.76, *p* = 0.017) were independent, robust prognostic factors for local tumour recurrence (Table 4; Figure 5). If combining T-grouping and mRNA expression of NG2/CSPG4, the probability to develop a secondary loco-regional lesion was further increased (*p* < 0.001; Table 3; Figure 4).

**Table 4:** **Multivariate analyses of the prognostic implication of altered PG expression for the different clinical outcomes** ^**1**^ **Estimated regression coefficient and confidence interval;**

Prognostic indicator/PG	Estimate^1^(95% CI)	SE^2^	*p value* ^3^	HR^4^	Clinical outcome
T classification					
T2^5^	0.359 (0.153/0.565)	0.105	0,012	1.432	Loco-regional relapse
T3	1.857 (1.556/2.158)	0.153		6.404	
T4	2.160 (1.956/2.364)	0.104		8.671	
T3-T4 *vs* T1-T2	1.850 (1.691/2.009)	0.081	0.001	6.360	Loco-regional relapse
NG2/CSPG4 mRNA^6^					
*De novo* expression *vs* no expression	1.911 (1.735/2.087)	0.090	0.017	6.760	Loco-regional relapse
Precancerous lesions					
Presence *vs* Absence	1.328 (1.184/1.471)	0.073	0.005	3.773	Lymphnodal metastases
SDC2 stroma					
Positive *vs* Negative	2.035 (1.885/2.184)	0.076	0.007	7.652	Lymphnodal metastases
SDC2 stroma					
Positive *vs* Negative	2.160 (2.022/2.298)	0.070	0.003	8.671	Disease-related deaths
N classification					
Positive *vs* Negative	1.477 (1.326/1.628)	0.077	0.012	4.380	Distant metastasis
N classification					
Positive *vs* Negative	1.089 (0.967/1.211)	0.062	0.005	2.971	Disease-related deaths
N classification					
Positive *vs* Negative	1.164 (1.003/1.325)	0.082	<0.001	3.203	Any of the clinical outcomes^7^
SDC1 mRNA					
↓ /= *vs* ↑	−1.460 (−1.612/-1.309)	0.077	0.013	0.232	Distant metastasis
SDC1 mRNA					
↓ /= *vs* ↑	−0.845 (−1.007/-0.684)	0.082	0.012	0.429	Any of the clinical outcomes
SDC1 mRNA up-regulation **+** NG2/CSPG4 mRNA d*e novo* expression **+** SDC2 stroma					
Single condition *vs* combination of all 3 conditions^8^	−1.924 (−2.088/-1.760)	0.084	0.002	0.146	Distant metastasis
	−1.302 (−1.459/-1.146)	0.080	0.004	0.272	Disease-related deaths
	−1.014 (−1.194/-0.833)	0.092	0.003	0.363	Any of the clinical outcomes

^1^Estimated regression coefficient and confidence interval;

^2^Standard error of estimated regression coefficient;

^3^*p* value <0,05 were considered to be significant;

^4^Hazard Ratio estimated from Cox proportional hazard regression model;

^5^Compared to T1 stage;

^6^PG transcript expression (↓, down-regulated; ↑, up-regulated; =, not changed; *De novo* expression, *de novo* expressed in comparison to a healthy mucosal tissues pool that was used as sample calibrator) could be grouped according to the trend of each PG gene in relation to the clinical outcomes;

^7^Is referred to a patient that had at least one of the other outcomes within the follow-up;

^8^Refers to the comparison between a situation in which *all three* indicated conditions were manifested (“*combination of all 3 conditions*”) versus either condition alone or the combination of *any two* conditions;

*Abbreviations*: CI, Confidence Interval; HR, Hazard Ratio; SE, standard error.

Significant correlations with post-surgical lymphnodal metastases were further disclosed between gender (*p* = 0.043), the presence of precancerous lesions (*p* = 0.003; Figure 4), the occurrence of lymphnodal infiltration at time of surgery (*p* = 0.001), stromal expression of SDC2 (*p* = 0.015; Figure 4) and altered GPC6 protein expression in tumour cells (*p* = 0.01; Table 3). As predictable, “sentinel lymphnodes” at diagnosis significantly influenced the later appearance of more prominent lymphnodal lesions, but this factor was not considered in the multivariate logistic regression model because of its unuttered prognostic implication. Multivariate analysis corroborated that the occurrence of precancerous lesions (HR, 3.773, *p* = 0.005), and more incisively the presence of SDC2 in the stromal compartment (HR, 7.652, *p* = 0.007), but not GPC6 expression or gender of the patient, were independent prognostic markers for post-surgery secondary infiltrations of lymphnodes (Table 4; Figure 5). If we then accounted for both a history of precancerous lesions and SDC2 stromal expression, the probability to develop post-surgical lymphnodal infiltration was significantly increased (*p* = 0.001; Table 3; Figure 4).

Contrary to the above associations, univariate logistic analyses revealed that the N classification , at time of surgical removal of the primary tumour mass, and up-regulated transcription of SDC1 (Figure 4) were significantly associated with the formation of distant metastases ( *p* = 0.016 for both correlations; Table 3). The frequency of GPC6 expression in neoplastic cells did not satisfy the limit of significance (*p* = 0.052), but could be a potentially interesting indicator to take into account in future investigations on larger cohorts of patients. Conversely, multivariate analyses reinforced the impact of N classification (HR, 4.38, *p* = 0.012) and down-regulated or unaltered SDC1 expression (HR, 0.232, *p* = 0.013) as independent factors predicting the formation of distant metastases, albeit with opposite trends, (Table 4; Figure 5). When we next considered the combination of the unfavourable conditions represented by lymphnodal infiltration and up-regulation of SDC1 transcription, we unfolded a significantly increased probability to develop distant metastases (*p* = 0.004; Table 3; Figure 4). Noteworthy was also the fact that 91% of patients with up-regulated SDC1 transcription that developed distant metastases within the follow-up period invariably succumbed the disease.

Advanced T classification (*p* = 0.038), T3-T4 grouping (*p* = 0.043), positive N classification (*p* < 0.001; Figure 4), up-regulation of SDC1 transcription (*p* = 0.036) and stromal expression of SDC2 or GPC1 (*p* = 0.001 and *p* = 0.012 respectively; Table 3, Figure 4) were also found to be strongly associated with disease-related death. Cervical lymphnodal involvement (HR, 2.971, *p* = 0.005) and, more markedly, synthesis of SDC2 in the stromal cells (HR, 8.671, *p* = 0.003), established two independent predictors of survival (Table 4; Figure 5). The combination of these two conditions further decreased the survival probability of the patients (*p* < 0.001, Table 3; Figure 4).

In the evaluation of situations in which patients presented at any of the mentioned clinical outcomes, we similarly found a tight correlation between advanced T classification (*p* = 0.018), T3-T4 grouping (*p* = 0.005), positive N classification (*p* < 0.001; Figure 4), up-regulation of SDC1 mRNA (*p* = 0.013; Figure 4) and SDC2 stromal reactivity (*p* = 0.002). Finally, in multivariate analyses, N involvement (HR, 3.203, *p* < 0.001) and down-regulation or unaltered SDC1 expression (HR, 0.429, *p* = 0.012), but not T classification or SDC2 detection in stromal cells, were independent factors with opposite trends for the prediction of poor prognosis (Table 4; Figure 5). Even in this case the combination of positive N classification and up-regulation of SDC1 mRNA expression significantly increased the probability of the patients to incur into a dismal disease course (*p* < 0.001;Table 3; Figure 4).

We finally evaluated the disease course in patients scoring positively for the 3 dismal prognostic indicators, i.e. *de novo* expression of NG2/CSPG4, stromal abundance of SDC2 and up-regulation of SDC1 mRNA, which, in an independent manner, associated with one or more of the adverse clinical outcomes. This conditions was found in 36 of the 173 patients (21%) and within this patient subgroup 17% and 25%, respectively, developed loco-regional secondary lesions or distant metastases. Lymphnodal metastasis was observed in 11% of the patients, whereas 33% of them succumbed to the disease. Survival analyses revealed a strong association between the PG pattern analyzed and the presence of distant metastases (*p* < 0.002), disease-related deaths (*p* = 0.004) and a cumulative bad prognosis (*p* < 0.004). The Cox regression model ascertained that patients not expressing simultaneously the three bad independent disease course markers resulted to have a best prognosis in terms of putative development of distant metastases (HR, 0.146, *p* = 0.002), for survival (HR, 0.272, *p* = 0.004) and for incurring into any of the unfavourable clinical events under consideration (HR, 0.363, *p* = 0.003) (Table 4; Figure 5).

## Discussion

Despite the relatively high incidence of oral cavity HNSCC, very few reliable prognostic and/or predictive molecular markers are currently available for the routine clinical management of the patients. In light of this deficiency, we have explored the possibility that variation in the expression of cell surface PGs, widely recognized to be key factors in the control of tumour progression [33,56-59], could afford more effective means of prognosticating patients affected by these tumours. Indeed, we find that, upon neoplastic transformation, epithelial cells of the oral cavity and oropharynx modify their transcriptional/translational rates of virtually *all* currently known cell surface PGs. This led us to conclude that transformation-dependent modulation of PG synthesis may be part of the globally altered pattern of gene expression in these cells, as well as contribute to the cancer cell’s acquisition of a defined repertoire a cell surface molecules capable of dictating their malignant behavior.

SDCs are widely recognized to undergo malignancy-associated changes in their expressions in several types of carcinomas, including those of thyroid, breast, colon, skin, stomach and urogenital tract, and SDC1 is recognized to be the best documented prognostic biomarker [39,41,43,40,46,60-64]. Its expression pattern frequently correlates with the differentiation status of the cells and thereby with their malignancy degree [47,54,65-68]. This characterizing trait of the SDC1 tumour-associated expression was corroborated here, along with its widespread distribution in neoplastic HNSCC lesions.

Although much less studied, SDC2 has also been reported to be associated with malignant carcinoma lesions in various anatomical sites/organs including head and neck [63,69,70]. In this study, transcription/translation of SDC2 was found to be more prominent in the intralesional stroma than in the neoplastic cells and, hence, the PG showed an expression pattern that was complementary to SDC1. Notably, SDC2 was also observed to be strongly enriched in neovascular structures, where it appeared to be associated with both endothelial cells and pericytes. Our present mapping study is the first to reveal a *de novo* expression of SDC3 and SDC4 in oral cavity HNSCC and the accumulation of SDC4 in areas of cell-cell contact [22,24,25] within such lesions. Intracellular abundance of SDCs in HNSCC cells may reflect the incapacity of the cells to complete the post-translational processing of these PGs and/or their transport and intercalation into the cell membrane, or an accentuated internalization and intracellular recycling process.

Several GPCs were also found to be misexpressed in oral cavity HNSCC lesions, albeit with frequencies that were generally lower than those seen for SDCs. GPC1, known to be highly expressed in pancreatic and breast carcinomas [32,33], was found to be the prevalent GPC of these lesions, alongside with GPC3, which has independently been reported to be up-regulated in several other tumour types and has a recognized value as prognostic factor and putative therapeutic target in hepatocellular carcinomas [34,35,37]. In this context, it is, however, worth noting that GPC3 has also been proposed to act as a potential tumour suppressor in certain neoplasia, showing a putative transformation-dependent silencing of the glypican [30,71-73].

When we applied univariate and multivariate meta-analytical methods to correlate the observed PG expression patterns with clinically relevant disease outcomes we unveiled striking associations. Appearance of NG2/CSPG4, a PG with a precedent prognostic impact in numerous solid tumours [74-82], was found to tightly correlate with loco-regional tumour recurrence and, hence, was disclosed to be the first ever to be described molecular relapse predictor in oral cavity HNSCC. Enhanced expression of GPC1 in the stromal compartment of these lesions also closely correlated with tumour recurrence and paralleled the more predictable prognostic implication of tumour staging. Beside its prognostic role in pancreatic cancer, there is currently no other indication that altered expression of GPC1 may influence the course of any tumour type. Another crucial finding of this study was the close association betweenSDC2 up-regulation in the intralesional stromal compartment and the overall survival of the patients carrying such SDC2-rich primary lesions. Even in this case, the present study provides the first evidence for such a prognostic relationship in any cancer type and, similarly to the potential of NG2/CSPG4, emphasizes that SDC2 may serve as a putative target for prevention and/or treatment of relapsing oral cavity HNSCC.

## Conclusion

The present study provides the first evidence that altered expression of cell surface-bound PGs is strongly links to the formation and progression of oral cavity HNSCC. Elective modulation of PG expression in primary oral cavity HNSCC lesions correlates, in a predictive manner, with several clinical outcomes and may therefore serve as an adjunct in the molecular diagnosis of these tumours. More specifically, enhanced expression of SDC2 in the tumour stroma significantly correlates with overall survival and is indicative of lymphonodal metastasis, whereas aberrant increased of SDC1 transcription is indicative of the presence of distant metastases. Strikingly, up-regulation of NG2/CSPG4 is tightly linked to loco-regional recurrence of the tumour, underscoring the potential of this biomarker to forcefully predict the clinical course of oral cavity HNSCC patients.

## Additional files

Additional file 1: Table S1. Patient demographic and clinic-pathological features.
Additional file 2: Figure S1. Overview of the distribution of clinical outcomes manifested by oral cavity HNSCC patients during the entire follow-up period subdivided in 6-months time intervals.
Additional file 3.
**Supplemental and detailed Materials and methods.**

Additional file 4: Table S2. Listing of the individual relative PG expression data.
Additional file 5: Figure S2. Representative immunostaining on healthy control tissue sections: GPC1 (**a**), GPC3 (b), GPC4 (c), GPC6 (d), NG2/CSPG4 (e), SDC1 (f), SDC2 (g), SDC3 (h) and SDC4 (i).
