# Supplementary material for: Proteoglycan-based diversification of disease outcome in head and neck cancer patients identifies NG2/CSPG4 and syndecan-2 as unique relapse and overall survival predicting factors
Source: BMC Cancer. 2015 May 3;15:352. doi: 10.1186/s12885-015-1336-4 (PMC4429505; doi:10.1186/s12885-015-1336-4)
Supplement: Additional file 1: Table S1. — Patient demographic and clinic-pathological features. [file 12885_2015_1336_MOESM1_ESM.docx]

**Supplementary Table 1.** *Patient demographic and clinic-pathological features^1^.*

| **Patient** | **Gender** | **Age** | **Tumor location** | **Smoke** | **Alcohol^2^** | **Familial cancer history^3^** | **Precancerous lesions^4^** | **Radiotherapy** | **D.D.** | **TNM** | **Lymphnode** | **L. R.** | **L. M.** | **D .M.** | **Death** |
| --- | --- | --- | --- | --- | --- | --- | --- | --- | --- | --- | --- | --- | --- | --- | --- |
| PR2 | F | 30 | OC | Yes | No | No | No | No | 3 | T1 | Negative | No | No | No | No |
| PR3 | M | 76 | OC | Yes | Yes | No | No | Yes |  | T4 | Positive | No | No | No | No |
| PR4 | F | 32 | OC | Yes | No | No | No | No | 2 | T1 | Negative | No | No | No | No |
| PR5 | M | 59 | OC | Yes | Yes | No | No | Yes | 3 | T4 | Positive | No | No | No | No |
| PR6 | F | 36 | OC | No | No | No | No | No |  | T1 | Negative | No | No | No | No |
| PR7 | M | 42 | OC | No | No | No | No | No | 3 | T2 | Negative | No | No | No | No |
| PR8 | M | 42 | OC | Yes | Yes | No | No | Yes | 2 | T4 | Positive | Yes | Yes | No | Yes |
| PR9 | M | 49 | OC | No | No | No | No | Yes |  | T4 | Positive | No | No | No | No |
| PR10 | M | 63 | OP | Yes | Yes | No | No | Yes | 3 | T4 | Positive | No | No | No | No |
| PR11 | M | 78 | OC | No | Yes | No | No | Yes | 2 | T4 | Positive | No | No | No | No |
| PR12 | F | 60 | OC | No | No | No | No | No | 1 | T1 | Negative | No | No | No | No |
| PR13 | M | 56 | OC | No | Yes | No | No | Yes | 3 | T4 | Positive | Yes | Yes | No | No |
| PR14 | M | 73 | OC | No | Yes | No | No | No | 1 | T2 | Negative | No | No | No | No |
| PR15 | M | 62 | OC | Yes | No | No | No | No | 2 | T1 | Negative | No | No | No | No |
| PR16 | F | 87 | OC | No | No | No | No | No | 2 | T4 | Negative | No | No | No | No |
| PR17 | F | 71 | OP | Yes | Yes | No | No | Yes | 3 | T4 | Positive | No | No | No | Yes |
| PR18 | F | 56 | OC | Yes | Yes | No | No | Yes | 1 | T2 | Positive | No | No | Yes | Yes |
| PR19 | M | 67 | OC | Yes | Yes | No | No | No | 1 | T2 | Negative | No | No | No | No |
| PR20 | F | 68 | OP | Yes | Yes | No | No | Yes | 3 | T4 | Positive | No | No | No | No |
| PR21 | M | 61 | OC | Yes | No | No | No | Yes | 3 | T4 | Positive | Yes | Yes | No | No |
| PR22 | M | 77 | OP | No | Yes | No | No | No | 3 | T2 | Negative | No | No | No | No |
| PR23 | M | 73 | OP | Yes | Yes | No | No | No | 3 | T2 | Negative | No | No | No | No |
| PR24 | M | 60 | OC | Yes | Yes | No | No | Yes | 3 | T4 | Positive | No | No | No | No |
| PR26 | F | 64 | OC | No | No | No | No | No | 3 | T1 | Negative | No | No | No | No |
| PR28 | F | 75 | OC | No | No | No | No | No | 3 | T1 | Negative | No | No | No | No |
| PR29 | F | 66 | OC | No | No | No | No | No |  | T1 | Negative | No | No | No | No |
| PR30 | M | 28 | OC | Yes | Yes | No | No | No |  | T2 | Negative | No | No | No | No |
| PR31 | M | 51 | OC | Yes | Yes | No | No | Yes | 3 | T2 | Positive | No | No | No | No |
| PR32 | F | 76 | OC | Yes | No | No | No | Yes | 3 | T2 | Positive | No | No | No | No |
| PR33 | M | 45 | OC | No | No | No | No | Yes | 3 | T4 | Negative | No | No | No | No |
| PR34 | F | 67 | OC | No | No | No | No | No | 3 | T2 | Negative | No | No | No | No |
| PR35 | F | 79 | OC | No | No | No | No | Yes | 2 | T3 | Negative | No | No | No | No |
| PR36 | M | 56 | OP | Yes | Yes | No | No | Yes | 2 | T2 | Positive | Yes | No | No | Yes |
| PR37 | F | 65 | OC | Yes | Yes | No | No | Yes | 3 | T3 | Positive | No | No | No | No |
| PR38 | M | 73 | OC | No | No | No | No | No | 3 | T2 | Negative | Yes | No | No | No |
| PR39 | F | 48 | OC | Yes | Yes | No | No | Yes | 2 | T2 | Positive | No | No | Yes | Yes |
|  |  |  |  |  |  |  |  |  |  |  |  |  |  |  |  |
| **Patient** | **Gender** | **Age** | **Tumor location** | **Smoke** | **Alcohol** | **Familial cancer history** | **Precancerous lesions** | **Radiotherapy** | **D.D.** | **TNM** | **Lymphnode** | **L. R.** | **L. M.** | **D .M.** | **Dead** |
| PR42 | M | 63 | OC | Yes | No | No | No | No | 3 | T1 | Positive | No | Yes | No | No |
| PR43 | F | 78 | OC | No | No | No | No | Yes | 3 | T2 | Positive | No | No | No | No |
| PR44 | F | 71 | OC | No | No | No | No | Yes | 2 | T4 | Positive | Yes | No | No | No |
| PR45 | M | 69 | OC | No | No | No | No | Yes | 3 | T3 | Positive | Yes | No | No | No |
| PR46 | F | 67 | OC | No | No | No | No | Yes | 3 | T2 | Negative | No | No | No | No |
| PR47 | M | 69 | OC | Yes | No | No | No | Yes | 3 | T3 | Positive | No | No | No | No |
| PR49 | M | 44 | OC | Yes | Yes | No | No | No | 3 | T1 | Negative | No | No | No | No |
| PR51 | F | 85 | OC | Yes | No | No | No | No | 3 | T1 | Negative | No | No | No | No |
| PR53 | M | 74 | OC | Yes | Yes | No | No | Yes | 3 | T2 | Negative | No | No | No | No |
| PR54 | M | 64 | OC | Yes | Yes | No | No | Yes | 3 | T1 | Positive | No | No | No | Yes |
| PR55 | F | 52 | OC | Yes | No | No | No | Yes | 3 | T1 | Negative | No | No | No | No |
| PR56 | M | 49 | OC | Yes | No | No | No | No | 2 | T1 | Negative | Yes | No | No | No |
| PR57 | F | 58 | OC | Yes | No | No | No | No | 2 | T1 | Negative | No | No | No | No |
| PR58 | F | 69 | OC | No | No | No | No | Yes | 3 | T4 | Positive | No | No | Yes | Yes |
| PR59 | M | 55 | OC | Yes | No | No | No | Yes | 3 | T1 | Negative | No | Yes | No | No |
| PR61 | F | 65 | OC | No | No | No | No | Yes | 3 | T1 | Negative | No | No | No | No |
| PR62 | M | 70 | OC | No | No | No | No | No | 3 | T1 | Negative | No | No | No | No |
| PR63 | M | 78 | OC | Yes | No | No | No | Yes | 3 | T2 | Positive | No | Yes | Yes | Yes |
| PR64 | M | 79 | OC | Yes | No | No | No | No | 2 | T1 | Negative | No | No | No | No |
| PR65 | F | 64 | OC | Yes | Yes | No | No | Yes | 3 | T2 | Negative | No | No | No | Yes |
| PR66 | F | 69 | OC | Yes | Yes | No | No | Yes | 3 | T4 | Negative | No | No | No | No |
| PR67 | M | 83 | OC | Yes | No | No | No | Yes | 3 | T4 | Negative | No | No | No | No |
| PR68 | F | 76 | OC | No | No | No | No | Yes | 3 | T2 | Positive | No | No | Yes | Yes |
| PR69 | M | 72 | OC | Yes | Yes | No | No | Yes | 3 | T1 | Positive | No | No | Yes | Yes |
| PR70 | F | 89 | OC | No | No | No | No | No | 3 | T1 | Negative | No | No | No | No |
| PR72 | M | 66 | OC | No | No | No | No | Yes | 3 | T2 | Negative | No | No | No | No |
| PR73 | F | 69 | OC | Yes | Yes | No | No | Yes | 3 | T4 | Negative | No | No | No | No |
| PR74 | M | 76 | OC | No | No | No | No | Yes | 3 | T1 | Positive | No | No | No | No |
| PR75 | M | 62 | OC | No | Yes | No | No | Yes | 3 | T2 | Positive | No | No | No | No |
| PR76 | M | 69 | OC | No | Yes | No | No | Yes | 3 | T4 | Negative | Yes | No | Yes | Yes |
| PR77 | F | 70 | OC | Yes | Yes | No | No | Yes | 3 | T4 | Positive | No | No | No | No |
| PR79 | M | 69 | OC | No | Yes | No | No | Yes | 3 | T2 | Positive | No | No | Yes | No |
| PR80 | F | 73 | OC | No | No | No | No | Yes | 3 | T2 | Positive | No | No | No | No |
| PR81 | F | 64 | OC | Yes | No | No | No | Yes | 2 | T4 | Positive | No | No | No | No |
| PR83 | M | 60 | OC | Yes | Yes | Yes | No | Yes | 3 | T4 | Positive | No | No | No | No |
| PR84 | M | 65 | OC | Yes | No | No | No | No | 3 | T2 | Negative | No | No | No | No |
|  |  |  |  |  |  |  |  |  |  |  |  |  |  |  |  |
| **Patient** | **Gender** | **Age** | **Tumor location** | **Smoke** | **Alcohol** | **Familial cancer history** | **Precancerous lesions** | **Radiotherapy** | **D.D.** | **TNM** | **Lymphnode** | **L. R.** | **L. M.** | **D .M.** | **Dead** |
| PR85 | M | 72 | OC | Yes | No | No | No | Yes | 3 | T2 | Positive | No | No | No | No |
| PR86 | F | 81 | OC | No | No | No | No | No | 2 | T2 | Negative | No | No | No | No |
| PR88 | M | 64 | OC | Yes | Yes | No | No | Yes | 3 | T3 | Positive | Yes | No | No | No |
| PR90 | M | 61 | OC | Yes | Yes | No | No | No | 3 | T2 | Positive | No | No | No | No |
| PR91 | F | 74 | OC | No | No | No | No | No | 3 | T2 | Negative | No | No | No | No |
| PR92 | M | 78 | OC |  |  | No | No | No | 3 | T1 | Negative | No | No | No | Yes |
| PR93 | M | 78 | OC | Yes | No | No | No | Yes | 3 | T1 | Positive | No | No | No | No |
| PR94 | F | 85 | OC | No | No | No | No | No | 3 | T1 | Negative | No | No | No | No |
| PR95 | F | 56 | OC | No | No | No | No | Yes | 3 | T2 | Positive | No | Yes | No | Yes |
| PR96 | F | 66 | OC | No | No | No | No | No |  | T1 | Negative | No | No | No | No |
| PR97 | F | 58 | OC | Yes | No | No | No | Yes | 3 | T4 | Positive | No | No | No | No |
| PR98 | F | 55 | OC | No | No | No | No | Yes | 3 | T4 | Positive | No | No | No | No |
| PR99 | M | 58 | OC | Yes | No | No | No | Yes | 3 | T1 | Negative | No | No | No | No |
| PR100 | M | 80 | OC |  |  | No | No | Yes | 3 | T2 | Positive | No | No | No | No |
| PR101 | F | 48 | OC | No | No | No | No | Yes | 2 | T4 | Positive | Yes | No | No | No |
| PR102 | F | 60 | OC | No | No | No | No | Yes | 3 | T2 | Positive | No | No | No | No |
| BB1 | F | 48 | OC | No | No | Yes | Yes | No | 2 | T1 | Positive | No | Yes | No | No |
| BB2 | M | 65 | OC | Yes | Yes | Yes | Yes | Yes | 2 | T1 | Negative | No | No | No | No |
| BB4 | F | 76 | OC | Yes | Yes | Yes | No | No | 2 | T2 | Negative | No | No | No | No |
| BB5 | M | 47 | OC | No | Yes | No | Yes | No | 3 | T2 | Positive | No | No | No | Yes |
| BB6 | M | 59 | OC | Yes | No | No |  | No | 2 | T1 | Negative | No | Yes | No | No |
| BB7 | M | 61 | OC+OP | No | Yes | No | Yes | Yes | 2 | T2 | Negative | No | No | No | No |
| BB8 | M | 59 | OP | No | Yes | No | No | No | 2 | T1 | Negative | No | No | No | No |
| BB10 | M | 26 | OC | No | No | No | Yes | No |  | T1 | Negative | No | No | No | No |
| BB11 | F | 61 | OC | No | No | No | Yes | No | 2 | T1 | Negative | No | No | No | No |
| BB12 | M | 61 | OC | Yes | Yes | No | Yes | Yes | 2 | T2 | Positive | No | No | No | No |
| BB14 | F | 73 | OC | Yes | Yes | Yes | Yes | No | 2 | T1 | Negative | Yes | No | No | No |
| BB15 | M | 65 | OC | Yes | Yes | No | Yes | Yes | 2 | T2 | Positive | No | No | No | No |
| BB16 | F | 56 | OC | Yes | No | No | No | No | 2 | T2 | Negative | No | No | No | No |
| BB17 | M | 54 | OC | Yes | Yes | No | Yes | Yes | 3 | T3 | Positive | No | No | No | No |
| BB18 | M | 57 | OC | Yes | Yes | No | Yes | Yes | 2 | T2 | Negative | No | Yes | Yes | Yes |
| BB19 | F | 55 | OC | Yes | No | No | Yes | No | 2 | T4 | Negative | No | No | No | No |
| BB20 | M | 61 | OC | Yes | No | No | No | No | 3 | T1 | Negative | No | No | No | No |
| BB21 | M | 54 | OC | No | No | Yes | Yes | No | 3 | T2 | Negative | No | No | No | No |
| BB22 | F | 70 | OC | No | Yes | Yes | Yes | Yes | 3 | T1 | Positive | No | No | No | No |
| BB23 | F | 59 | OC | Yes | Yes | No | No | No | 2 | T4 | Positive | No | No | Yes | Yes |
| BB24 | F | 78 | OC | No | No | Yes | Yes | No | 2 | T4 | Negative | No | No | No | No |
| **Patient** | **Gender** | **Age** | **Tumor location** | **Smoke** | **Alcohol** | **Familial cancer history** | **Precancerous lesions** | **Radiotherapy** | **D.D.** | **TNM** | **Lymphnode** | **L. R.** | **L. M.** | **D .M.** | **Dead** |
| BB26 | M | 89 | OC | Yes | Yes | Yes | Yes | No | 3 | T4 | Negative | No | No | No | Yes |
| BB27 | F | 89 | OC | Yes | Yes | Yes | Yes | No | 3 | T4 | Negative | No | Yes | No | No |
| BB28 | M | 61 | OP | Yes | No | No | Yes | Yes | 3 | T2 | Positive | No | No | No | No |
| BB29 | M | 73 | OC | Yes | Yes | No | Yes | Yes | 3 | T4 | Positive | No | Yes | No | Yes |
| BB30 | M | 53 | OC | Yes | No | No | Yes | No | 1 | T4 | Negative | No | No | No | No |
| BB31 | F | 60 | OC | No | Yes | No | Yes | No | 1 | T1 | Negative | No | No | No | No |
| BB32 | M | 67 | OC | Yes | Yes | No | Yes | No | *in situ* | T1 | Negative | No | No | No | No |
| BB33 | F | 62 | OC+OP | Yes | No | No | Yes | Yes | 2 | T4 | Negative | Yes | No | No | No |
| BB34 | F | 60 | OC | Yes | No | No | Yes | No |  | T1 | Negative | No | No | No | No |
| BB35 | F | 72 | OC | Yes | No | No | Yes | No | 2 | T1 | Negative | No | No | No | No |
| BB36 | M | 56 | OC | Yes | Yes | No | Yes | Yes | 3 | T4 | Positive | No | Yes | No | No |
| BB37 | F | 59 | OC | Yes | Yes | No | Yes | Yes | 2 | T4 | Positive | No | No | No | No |
| BB38 | M | 58 | OC | Yes | Yes | No | No | No | 2 | T1 | Negative | No | No | No | No |
| BB40 | M | 71 | OC | Yes | Yes | No | Yes | Yes | 2 | T4 | Negative | No | Yes | Yes | Yes |
| BB41 | M | 58 | OC | Yes | Yes | No | Yes | No | 2 | T2 | Negative | No | No | No | No |
| BB42 | M | 71 | OC | Yes | Yes | Yes | Yes | No | 2 | T2 | Negative | No | No | No | No |
| BB43 | M | 64 | OP | No | No | No | No | Yes | 3 | T1 | Positive | No | No | No | No |
| BB44 | M | 44 | OC | Yes | Yes | No | Yes | Yes | 3 | T3 | Positive | No | Yes | No | No |
| BB45 | F | 60 | OC | No | No | Yes | Yes | No |  | T2 | Negative | No | No | No | No |
| BB46 | M | 72 | OC | Yes | No | Yes | No | No | 2 | T3 | Negative | No | No | No | No |
| BB47 | M | 61 | OC | No | No | Yes | Yes | Yes | 3 | T2 | Positive | No | No | No | No |
| BB48 | F | 50 | OC | No | No | No | Yes | Yes | 3 | T2 | Positive | No | No | No | No |
| BB49 | M | 80 | OP | Yes | Yes | Yes | No | No | 2 | T1 | Negative | No | No | No | No |
| BB50 | M | 40 | OP | Yes | Yes | No | Yes | Yes | 1 | T2 | Positive | No | No | No | No |
| BB51 | F | 59 | OC | Yes | Yes | No | Yes | Yes | 2 | T2 | Positive | No | No | Yes | No |
| BB52 | F | 93 | OC | No | No | Yes | Yes | No | 1 | T2 | Negative | No | No | No | No |
| BB53 | F | 56 | OC | No | No | No | Yes | No | 2 | T1 | Negative | No | No | No | No |
| BB56 | M | 68 | OC+OP | Yes | Yes | Yes | Yes | Yes | 2 | T2 | Positive | No | Yes | No | Yes |
| BB58 | F | 89 | OC | No | No | Yes | Yes | No | 1 | T2 | Negative | No | No | No | No |
| BB60 | F | 70 | OC | No | Yes | Yes | Yes | Yes |  | T2 | Positive | Yes | Yes | Yes | Yes |
| BB65 | F | 85 | OC | No | No | No | No | Yes |  | T1 | Positive | No | No | No | No |
| BB68 | M | 79 | OC | No | Yes | No | Yes | Yes |  | T3 | Positive | Yes | Yes | No | Yes |
| BB70 | M | 68 | OC | Yes | Yes | No | Yes | Yes |  | T2 | Positive | Yes | No | No | Yes |
| BB71 | M | 72 | OC | No | Yes | No | Yes | Yes |  | T3 | Positive | No | Yes | No | Yes |
| BB72 | M | 80 | OC | Yes | Yes | No | No | Yes |  | T3 | Positive | No | No | No | Yes |
| BS003 | F | 82 | OC | No | No | No | Yes | No | 1 | T2 | Negative | Yes | No | No | No |
| BS005 | M | 77 | OC | Yes | Yes | No | Yes | No | *in situ* | T2 | Negative | No | No | No | No |
| **Patient** | **Gender** | **Age** | **Tumor location** | **Smoke** | **Alcohol** | **Familial cancer history** | **Precancerous lesions** | **Radiotherapy** | **D.D.** | **TNM** | **Lymphnode** | **L. R.** | **L. M.** | **D .M.** | **Dead** |
| BS006 | F | 41 | OC | Yes | Yes | Yes | No | No | 2 | T4 | Negative | No | No | No | No |
| BS007 | M | 65 | OP | Yes | Yes | Yes | No | Yes |  | T3 | Positive | No | No | Yes | Yes |
| BS008 | M | 30 | OC | Yes | Yes |  | No | No | 1 | T2 | Negative | No | No | No | No |
| BS009 | F | 85 | OC | Yes | No | No | No | No | 1 | T4 | Negative | Yes | No | No | Yes |
| BS010 | M | 69 | OC | Yes | Yes | No | No | No | 1 | T4 | Negative | No | No | No | No |
| BS011 | F | 49 | OC | Yes | Yes | No | No | No | *in situ* | T3 | Negative | No | No | No | No |
| BS013 | M | 66 | OC | Yes | Yes | No | No | Yes | 2 | T4 | Positive | Yes | No | No | Yes |
| BS018 | F | 89 | OC | No | No | No | Yes | No | 2 | T1 | Negative | No | No | No | No |
| BS019 | M | 42 | OC | Yes | Yes | Yes | No | No | 2 | T3 | Negative | No | No | No | No |
| BS020 | F | 70 | OC | Yes | Yes | No | No | Yes | 1 | T2 | Positive | No | No | No | No |
| BS021 | M | 55 | OC | Yes | Yes | No | No | No | 1 | T2 | Negative | No | No | No | No |
| BS024 | M | 32 | OC | Yes | Yes | No | No | No | 1 | T1 | Positive | No | No | No | No |
| BS025 | M | 60 | OC | Yes | Yes | No | No | No | 1 | T1 | Negative | No | No | No | No |
| BS026 | M | 42 | OC | No | Yes | Yes | No | No | 1 | T2 | Negative | No | No | No | No |
| BS027 | F | 47 | OP | Yes | Yes | Yes | No | Yes | 3 | T4 | Positive | No | No | No | No |
| BS029 | M | 64 | OC | Yes | Yes | No | No | Yes | 2 | T4 | Positive | No | No | No | No |
| BS030 | M | 77 | OC | Yes | Yes | No | No | No |  | T2 | Negative | No | No | Yes | Yes |
| BS032 | M | 61 | OC | Yes | Yes | No | No | Yes | 2 | T4 | Negative | No | No | No | No |
| BS033 | M | 61 | OC | No | No | Yes | Yes | No | 2 | T2 | Negative | No | No | Yes | No |
| BS034 | M | 79 | OC | Yes | Yes | Yes | No | No | 1 | T4 | Negative | Yes | No | No | Yes |
| BS035 | M | 83 | OC | No | No | No | No | No | 1 | T1 | Negative | No | No | No | No |
| BS036 | M | 70 | OC | Yes | Yes | No | No | Yes | 2 | T4 | Positive | No | No | Yes | Yes |
| BS037 | M | 27 | OC | Yes | Yes | No | No | No |  | T3 | Negative | Yes | No | No | No |
| BS038 | F | 73 | OC | Yes | No | No | Yes | No |  | T1 | Negative | No | No | No | No |
| BS039 | M | 53 | OC | Yes | Yes | Yes | Yes | No |  | T3 | Negative | No | No | No | No |
| BS041 | F | 79 | OC | No | No |  | Yes | No |  | T1 | Negative | No | No | No | No |
| BS042 | F | 62 | OP | Yes | Yes | No | No | Yes |  | T3 | Positive | No | No | No | No |

*Abbreviations*: F, female; M, male; OC, oral cavity; OP, oropharynx; D.D., degree of differentiation; L.R., loco-regional relapse; L.M., lymphnode metastasis; D.M., distant metastasis.

^1^Post-surgical follow-up period ranging from 6 to 24 months; ^2^excessive alcohol consumption was based upon self-provided information; ^3^familial cancer history is referred to non-related OSCC tumor(s)
