# Supplementary material for: Proteoglycan-based diversification of disease outcome in head and neck cancer patients identifies NG2/CSPG4 and syndecan-2 as unique relapse and overall survival predicting factors
Source: BMC Cancer. 2015 May 3;15:352. doi: 10.1186/s12885-015-1336-4 (PMC4429505; doi:10.1186/s12885-015-1336-4)
Supplement: Additional file 2: Figure S1. — Overview of the distribution of clinical outcomes manifested by oral cavity HNSCC patients during the entire follow-up period subdivided in 6-months time intervals. [file 12885_2015_1336_MOESM2_ESM.docx]

**Supplemental Fig. 1.** Overview on distribution of patients outcomes from the beginning up to the end of follow-up subdivided in 6-months periods.

| Number of patients |
| --- |
