# Supplementary material for: Proteoglycan-based diversification of disease outcome in head and neck cancer patients identifies NG2/CSPG4 and syndecan-2 as unique relapse and overall survival predicting factors
Source: BMC Cancer. 2015 May 3;15:352. doi: 10.1186/s12885-015-1336-4 (PMC4429505; doi:10.1186/s12885-015-1336-4)
Supplement: Additional file 3. — Supplemental and detailed Materials and methods. [file 12885_2015_1336_MOESM3_ESM.docx]

**Supplemental data**

**Materials and Methods**

***Patients*** (full text)

The present study was conducted with full approval of the Human Research Ethics Committees of the involved institutions. Patients from whom surgical specimens were obtained were treated by head and neck surgeons from the Polyclinics S. Orsola-Malpighi and Bellaria Hospital, University of Bologna, and the Maxillo-Facial Surgery Division, Department of Head and Neck Surgery, University of Parma, who adopted analogous surgical procedures. At the same institutions normal epithelia of the oral cavity obtained during tooth extraction procedures were also collected as a control/reference tissue for molecular analyses. Tumors were diagnosed at the Departments of Surgical Pathology of the Universities of Bologna at Bellaria Hospital and Parma and the Bellaria Hospital. Selection criteria included derivation from primary HNSCC (as assessed by pre-operative biopsy), lack of previous radio- or chemo-treatments, and availability of fresh-frozen tissue for molecular analyses. A total of 173 surgical specimens of primary HNSCC (assessed by pre-operative biopsies), obtained from 99 male and 74 female patients of 26 to 93 years of age (mean 63.4±14), were collected after informed consent (***Supplemental Table 1, Supplemental Fig. 1***). Patients were referred to adjuvant radiation therapeutic treatment according to the guidelines defined by the National Comprehensive Cancer Network (NCCN) Clinical Practice (Version 2.2014; www.nccn.org). Smoke and/or alcohol consumption was admitted by 123 of the patients, among which 105 of them were habitual smokers, 88 referred regular alcohol consumption and 72 were both smokers and drinkers. The pathological examination of the surgical specimens revealed cumulatively well, moderately and poorly differentiated invasive carcinomas in 20, 50 and 78 of the cases, respectively. Based upon the 2010 TNM staging system (Edge et al, 2010) cases were classified as follows: 51 as pT1, 60 as pT2, 17 as pT3, and 45 as pT4. Lymphnodal metastases were detected at the time of diagnosis in 76 of the patients. Within the post-surgical follow-up period ranging from 6 to 24 months, 21 of the patients manifested local tumor recurrence, with 5 of these presenting concomitant lymphnodal metastases, and at the end of the observation period 32 patients had succumbed from the disease, and 93 patients were alive and free of tumor recurrence and/or metastasis. Healthy specimens and 119 neoplastic specimens, sampled as fresh tissue, were processed for molecular analyses according to the following scheme: each biopsy was dissected in three 1.5 cm-thick cubes, immersed into RNAlater Solution (Ambion) and frozen at -80°C. One of the three samples was used to extract total RNA for qPCR analyses, whereas the other two samples were used to create a tissue bank. The remaining part of these 119 samples and the other 54 tumor samples were fixed in formalin and paraffin-embedded according to routine procedures. Diagnoses and tumor staging were based upon the guidelines set by the World Health Organization (WHO) and the American Joint Committee on Cancer (AJCC). In TMAs (*see below*), 3 of the cases were composed only of *in situ* carcinoma, while the other 160 cases were diagnosed as invasive carcinomas. A total of 109 surgical specimens were analyzed at both transcriptional and protein level, whereas 10 specimens were evaluated only for PG mRNA expressions because the archival material was not sufficient for been included in TMA constructions. For 54 of the patients for which specimens were available for immunohistochemical analyses, it was not possible to obtain fresh material and, hence, these cases were only examined by immunohistochemistry.

***RNA extraction and qPCR*** (full text)

Total RNA from each biopsy was extracted using Trizol® according to the manufacturer’s instructions and in combination with Qiagen RNAeasy Mini Kit (Qiagen). RNA integrity and was verified using Thermo Scientific NanoDrop^TM^ spectrophotometers and micro-electrophoresis on a Bioanalyzer 2010 instrument (Agilent). Total RNA (1μg) was reverse-transcribed with the QuantiTect® Reverse Transcription Kit (Qiagen) according to the manufacturer’s instructions and cDNA was quantified. Each TaqMan Low Density Array was designed for quantification of the human PG homologues and were run using the TaqMan® technology based upon the fluorogenic 5’ nuclease assay. The assays were chosen among the TaqMan Gene Expression Assay library (**GPC1/**glypican-1, Hs00157805_m1; ***GPC2/****glypican-2*, Hs00415099_m1; ***GPC3/****glypican-3*, Hs00170471_m1; ***GPC4/****glypican-4*, Hs00155059_m1; ***GPC5/****glypican-5*, Hs00270114_m1; ***GPC6/****glypican-6*, Hs00170677_m1; ***SDC1/****syndecan-1*, Hs00896423_m1; ***SDC2/****syndecan-2*, Hs00299807_m1; ***SDC3/****syndecan-3*, Hs00206320_m1; ***SDC4/****syndecan-4*, Hs00161617_m1; and ***CSPG4/****NG2*, Hs00426981_m1) and the cards were run on a ABI PRISM 7900 HT Fast Real-Time PCR System (Applied Biosystems Inc., Foster City, CA, USA). Gene expression was normalized to the endogenous housekeeping gene 18S rRNA and to a pool of RNAs extracted from 5 normal epithelia that was adopted as a sample calibrator. An equal amount of input RNA (10 ng) was used per reaction and loaded in one of the eight sample-loading port of the card. For data analysis we used the RQ Manager software provided by Applied Biosystem Inc. Changes in gene expression levels were calculated using the “relative quantification method", which is based upon comparison of the expression levels of a target gene versus a reference gene, normally a housekeeping gene, and for each sample a further comparison with a sample calibrator. In our qPCR analyses we arbitrarily decided to consider reliable values lower than 35 Ct, as determined by raw data amplification, while those above 35 Ct were considered as “undetermined”, in accordance to criteria previously proposed by Lϋ et al. (Lϋ et al, 2008). Validation of our qPCR approach was provided by the finding that 5 genes with low Ct values (<35 cycles) had lower mean ΔCt variances (0.17388±0.16091) between intra-plate duplicates than 6 genes with high Ct values (>35 cycles; p<0.00223) that had high mean ΔCt variances (2.68343±1.67588). Furthermore, 6 genes examined in 9 plate replicates with low Ct values (<35 cycles) had lower mean ΔCt variances (0.23631±0.07109) than others 6 genes of the same replicates that had high mean ΔCt variances (1.354681±1.533506) with high Ct values (>35 cycles; p<0.05). To visualize the obtained expression profiles, we used heatmap graphing. Relative gene expression fold-change are expressed as 2^^-ΔΔCt^ value and they are transformed in Log2(2^^-ΔΔCt^) to generate the heat map by [EPCLUST](http://www.bioinf.ebc.ee/EP/Docs/EPCLUST/)-Expression Profile data CLUSTering and analysis software ([www.bioinf.ebc.ee/EP/EP/EPCLUST/](http://www.bioinf.ebc.ee/EP/EP/EPCLUST/)) (Brazma et al, 2000) applying average linkage UPGMA (Unweighted Pair Group Method with Arithmetic Mean) as clustering method and correlation measure based distance (uncentered) to measure the distance between cluster. The data presented herein have been deposited in NCBI's Gene Expression Omnibus (Barrett et al, 2009) and are accessible through GEO Series accession number GSE33788 (http://www.ncbi.nlm.nih.gov/geo/query/acc.cgi?acc=GSE33788).

***Tissue microarray (TMAs) construction*** (full text)

Each tumor tissue block containing representative areas of the neoplastic lesion was selected for TMA construction that was accomplished following a previously described procedure (Zimpfer et al, 2007; Tzankov et al, 2003). Briefly, haematoxylin/eosin-stained sections were obtained from paraffin blocks and used to define diagnostic areas. Subsequently, representative 0.6 mm-wide cores were dissected out from each tissue block and inserted into a grid pattern of a recipient paraffin block using a tissue arrayer (MTA-1, Beecher Instruments Inc.WI, USA). Cases were considered representative when at least 50% of the section was composed of neoplastic cells. For each case, the core portion of the section with the highest percentage of tumor cells was used for analysis.

***Immunohistochemistry*** (full text)

Sections (4 µm) were cut from TMA and stained with the following antibodies: anti-GPC1 MAB2600 (clone 4D1, Millipore Corporation, MA, USA) and anti-GPC1 cod. NBP1-89759 (Novus Biologicals, LLC, CO, USA), anti-GPC3 cod. B0025R (clone 1G12, BioMosaic Inc., VT, USA), anti-GPC4 cod. AH1008.2 (aa 54-66 serum peptide, Immundiagnostik AG, Germany), anti-GPC6 cod. HPA017671 (pAb, Sigma-Aldrich Co. LLC, MO, USA), anti-SDC1/CD138 cod. M7228 (clone MI15, Dako Denmark A/S, Denmark), anti-SDC2 MAB29651 (clone 305507, R&D Systems, MN, USA), anti-SDC2 cod. sc-73516 (1F10/b8) and anti-SDC2 cod. sc-73517 (2H7/G11, Santa Cruz Biotechnology, Inc., CA, USA), anti-SDC3 cod. HPA017087 (pAb, Sigma-Aldrich Co. LLC, MO, USA), anti-SDC4 cod. ab24511 (pAb, Abcam plc., Cambridge, UK) and anti-SDC4 cod. sc-15350 (pAb H-140, Santa Cruz Biotechnology, Inc., CA, USA), anti-NG2/CSPG4 HPA002951 (pAb, Sigma-Aldrich Co. LLC, MO, USA), anti-NG2/CSPG4 cod. 2166-G4-B9, cod. 2172-D12, cod. D2, cod. 2164-h5-b9, cod. 2164-d4-a6, cod. 2161-f9-c2, cod. 2166-G4, cod. 2171-B12, cod. 2164-H5-D8, cod. 2164-H5-E11, cod. 2164-H5, cod. 2172-B12-C10, cod. 2172-B12-F10 and cod. 2172-F11-G2. All antibodies were tested on tissues of known positivity served as positive controls; negative controls consisted in omission of the primary antibody: anti-GPC1 on human mammary tissue, anti-GPC3 on human hepatocarcinoma section, anti-GPC4 on human fetal nervous and kidney tissues, anti-GPC6 on human colon tissue, anti-SDC1 on myeloma sample, anti-SDC3 on human lymphnode, anti-NG2/CSPG4 on melanoma and high-grade glioma section. Simultaneous dewaxing and antigens unmasking was perfomed by incubation with the solutions W-Cap TEC buffer pH 6.0, or W-Cap TEC buffer pH 8.0 (Bio-Optica, Milan, Italy), for 25 minutes at 98° C. The endogenous peroxidase activity was blocked by incubation for 10 min minutes with H_2_O_2_ (3% in H_2_O), followed by washing in distilled water and incubation for 5 min with Blocking Solution (LabVision, Fremont, CA, USA) to further saturate non-specific binding sites (both incubations were carried out at room temperature). Primary monoclonal antibodies were applied on sections for 60 min at room temperature followed by washing and chromogenic detection using the UltraVision Detection System (LabVision, Fremont, CA, USA) involving incubation with Antibody Enhancer for 20 min and with HRP-Polymer for 30 min. Finally, DAB chromogen (Dako, Carpenteria, CA, USA) was applied for 3-5 min and sections were counterstained with hematoxylin. Relative antigen expression was assessed semi-quantitatively according to the arbitrary scoring: “-” = no positive cells were detected, “+” <10% of cells were positive, ≥10% “++” <50% of cells were positive, ≥50% “+++” <90% of cells were positive, and “++++” ≥90% of cells were positive.

***Statistical and bioinformatic analyses*** (full text)

Demographic data (sex and age), risk factors data associated to HNSCC (smoking and alcohol intake history, familial history of cancers, presence of precancerous lesions), clinical data (anatomical primary localization, T-score, lymphnode at appearance, tumor differentiation degree), gene expression data and immunohistochemical results for GPC1-6, SDC1-4 and NG2/CSPG4 were analyzed for their relationship with the following patients outcomes: loco-regional recurrence, lymphnodal metastasis, distant metastasis, survival and combined endpoints. Estimation of influence of each variable considered on the 5 patient’s outcomes were analyzed independently with both the Log-rank and Wilcoxon’s rank test. Survival rate was estimated using the Kaplan-Meier method from the time of surgery to the end of the follow-up. Univariate and stepwise multivariate logistic regression analyses were used to extract a parsimonious set of independent variables for each of the five outcomes selected. Predictors that were not found to be statistically significant by univariate analysis in at least one of the two statistical test applied were not included in the following Cox’s multivariate proportional hazards regression method. The levels of significance in the multivariate analysis were calculated by the likelihood ratio test. All analyses were performed using the Statgraphics Centurion XVI software (StatPoint Technologies, Inc, Virginia, USA). In all analyses, P values <0.05 were considered to be significant.

**REFERENCES to Supplemental data**

Edge S, Byrd D, Compton C et al. Breast AJCC cancer staging manual. 7th ed. New York: Springer-Verlag 2010: 347–369.

Lü B, Xu J, Chen J et al. [TaqMan low density array is roughly right for gene expression quantification in colorectal cancer.](http://www.ncbi.nlm.nih.gov/pubmed/18179774) Clin Chim Acta 2008; 389: 146-151.

Brazma A. and Vilo J. Gene expression data analysis. FEBS Lett 2000; 480: 17-24.

Barrett T, Troup DB, Wilhite SE et al. [NCBI GEO: archive for high-throughput functional genomic data.](http://www.ncbi.nlm.nih.gov/pubmed/18940857) Nucleic Acids Res 2009; 37(Database issue): D885-890.

Zimpfer A, Schonberg S, Lugli A et al. Construction and validation of a bone marrow tissue microarray. J Clin Pathol 2007; 60: 57-61.

Tzankov A, Pehrs AC, Zimpfer A, Ascani S, Lugli A, Pileri S, Dirnhofer S. [Prognostic significance of CD44 expression in diffuse large B cell lymphoma of activated and germinal centre B cell-like types: a tissue microarray analysis of 90 cases.](http://www.ncbi.nlm.nih.gov/pubmed/14514777) J Clin Pathol 2003; 56:747-752.
