# Supplementary material for: Proteoglycan-based diversification of disease outcome in head and neck cancer patients identifies NG2/CSPG4 and syndecan-2 as unique relapse and overall survival predicting factors
Source: BMC Cancer. 2015 May 3;15:352. doi: 10.1186/s12885-015-1336-4 (PMC4429505; doi:10.1186/s12885-015-1336-4)

**Supplemental Fig. 2.** Representative images of used PG antibodies staining on paraffin-embedded healthy control tissue sections: GPC1 (a), GPC4 (c) and SDC1 (f) positive staining; NG2/CSPG4 showed an immunoreaction in basal cells (e); SDC2 reacts just in the well of vessels (g), while GPC3 (b), GPC6 (d), SDC3 (h) and SDC4 (i) were not expressed


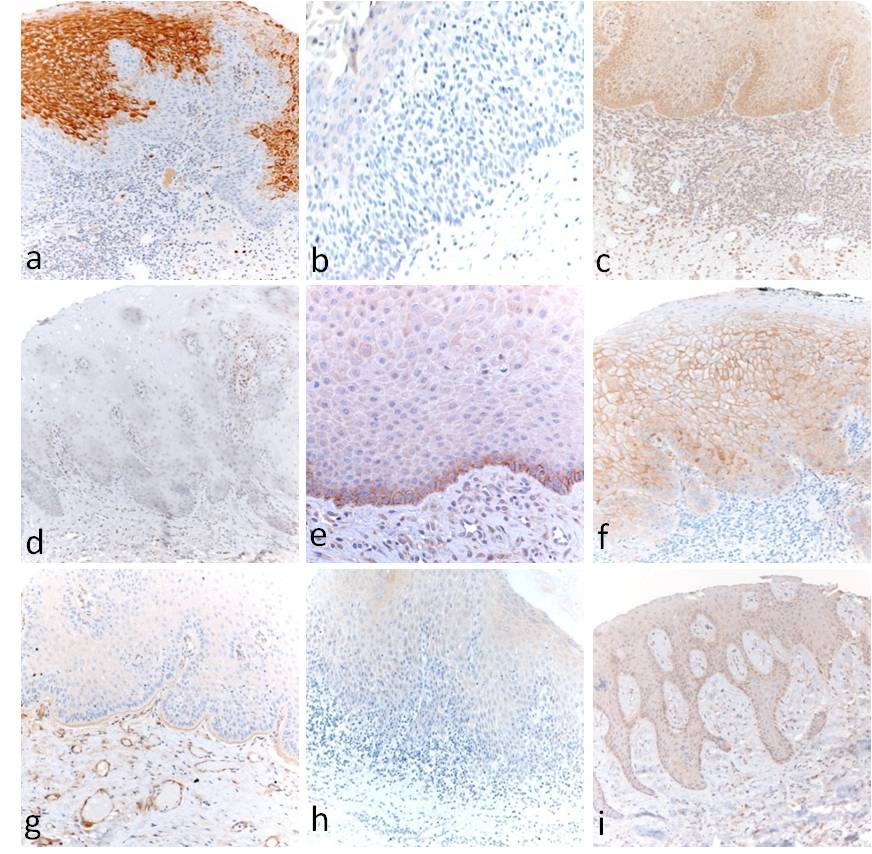

Supplement: Additional file 5: Figure S2. — Representative immunostaining on healthy control tissue sections: GPC1 (a), GPC3 (b), GPC4 (c), GPC6 (d), NG2/CSPG4 (e), SDC1 (f), SDC2 (g), SDC3 (h) and SDC4 (i). [file 12885_2015_1336_MOESM5_ESM.docx]
